# Naturally occurring quercetin and myricetin as potent inhibitors for human ectonucleotide pyrophosphatase/phosphodiesterase 1

**DOI:** 10.1038/s41598-023-50590-7

**Published:** 2024-01-02

**Authors:** Peeradon Duangiad, Bodee Nutho, Thawatchai Chaijarasphong, Noppawan Phumala Morales, Thunyarat Pongtharangkul, Itaru Hamachi, Akio Ojida, Jirarut Wongkongkatep

**Affiliations:** 1 Department of Biotechnology, Faculty of Science, Mahidol University, Rama 6 Road, Bangkok, 10400 Thailand; 2 Department of Pharmacology, Faculty of Science, Mahidol University, Rama 6 Road, Bangkok, 10400 Thailand; 3 Department of Synthetic Chemistry and Biological Chemistry, Graduate School of Engineering, Kyoto University, Katsura, Nishikyo-Ku, Kyoto, 615-8510 Japan; 4 Graduate School of Pharmaceutical Sciences, Kyushu University, 3-1-1, Maidashi, Higashi-Ku, Fukuoka, 812-8582 Japan

**Keywords:** Computational biology and bioinformatics, Drug discovery, Health care

## Abstract

Ecto-nucleotide pyrophosphatases/phosphodiesterases 1 (ENPP1) is a key enzyme in purinergic signaling pathways responsible for cell-to-cell communications and regulation of several fundamental pathophysiological processes. In this study, Kyoto Green, a rapid chemical sensor of pyrophosphate, was employed to screen for effective ENPP1 inhibitors among five representative flavonoids (quercetin, myricetin, morin, kaempferol, and quercetin-3-glucoside), five nucleosides (adenosine, guanosine, inosine, uridine, and cytidine), and five deoxynucleosides (2′- and 3′-deoxyadenosine, 2′-deoxyguanosine, 2′-deoxyinosine, and 2′-deoxyuridine). Conventional colorimetric, fluorescence, and bioluminescence assays revealed that ENPP1 was effectively inhibited by quercetin (*K*_i_ ~ 4 nM) and myricetin (*K*_i_ ~ 32 nM) when ATP was used as a substrate at pH 7.4. In silico analysis indicated that the presence of a chromone scaffold, particularly one containing a hydroxyl group at the 3′ position on the B ring, may promote binding to the active site pocket of ENPP1 and enhance inhibition. This study demonstrated that the naturally derived quercetin and myricetin could effectively inhibit ENPP1 enzymatic activity and may offer health benefits in arthritis management.

## Introduction

Purinergic signaling pathways are universal systems of cell-to-cell communication that regulate several fundamental pathophysiological processes such as immunity, inflammation, cancer, tissue homeostasis, wound healing, and neurodegeneration^1,2^. As extracellular nucleosides and nucleotides serve crucial signaling roles in these pathways, their concentrations are subject to tight regulation by various enzymes in the ecto-nucleotidase family, including ecto-nucleoside triphosphate diphosphohydrolases (NTPDases, EC 3.6.1.5), ecto-nucleotide pyrophosphatases/phosphodiesterases (NPPs, EC 3.6.1.9 and EC 3.1.4.1), tissue-nonspecific alkaline phosphatases (TNAPs, EC 3.1.3.1), and ecto-5′-nucleotidase (eN, CD73, EC 3.1.3.5)^3^. In human, NPPs can be divided into seven types, ENPP1-7, that share structural similarities but vary in substrate preference. While ENPP1, ENPP3, ENPP4 and ENPP5 hydrolyze nucleotides^2,4^, others hydrolyze NAD^+^, FAD, UDP-sugars, cyclic (di-) nucleotides, and dinucleoside polyphosphates. The most notable member of human NPPs is ENPP1, which is highly expressed in osteoblasts and chrondrocytes, and plays a crucial role in bone mineralization^5^. Moreover, high activity of ENPP1 has also been linked to crystal deposition diseases such as soft tissue calcification and pseudogout, as well as multiple types of cancer, including lung, breast, and ovarian cancer^6,7^. The association with these diseases make ENPP1 an attractive target for immunotherapy of various cancers and tissue calcification^8–16^.

Several synthetic compounds have been reported as potent inhibitors of ENPP1, especially those containing naphthalene, pyrimidine, purine, quinazoline, quinolone and thioguanine scaffolds^3,4,9–15^. However, naturally sourced ENPP1 inhibitors are still scarce, with myricetin as the only known natural product capable of inhibiting ENPP1^16^. Therefore, the untapped potential of natural sources as a rich reservoir of ENPP1 remains an area that merits further investigation. Myricetin (3,5,7,3′,4′,5′-hexahydroxyflavone) and quercetin (3,5,7,3′,4′-pentahydroxyflavone) are common plant-derived flavonoids recognized for their nutraceutical properties and health benefits^17,18^. Abundant sources of quercetin include onions, apples, strawberries, teas, and coffees, while myricetin can be found in berries, vegetables, teas, and wines^19^. Myricetin, quercetin, as well as morin (3,5,7,2′,4′-pentahydroxyflavone), which is an isomer of quercetin, and kaempferol (3, 4′,5,7 -tetrahydroxyflavone), share a common flavone scaffold, each differing in positions and/or numbers of hydroxyl group(s)^19,20^. In terms of health benefits, quercetin has been shown to exhibit a wide range of pharmacological activities, including anti-hyperuricemia, antioxidant, anti-inflammation, as well as amelioration of metabolic syndromes and cardiovascular diseases, which are often comorbid with hyperuricemia and gout^18^. It is also a potential alternative for gout therapy owing to its reversible inhibition of xanthine oxidase, which catalyzes the formation of urate and superoxide radicals^21^. Nonetheless, it is unknown whether quercetin is capable of inhibiting ENPP1, a property that would hold great therapeutic potential for pseudogout, soft tissue calcification and various cancers.

As ENPP1 is capable of hydrolyzing artificial phosphoric acid esters such as *p*-nitrophenyl 5′-thymidine monophosphate (*p*-Nph-5′-TMP), it is possible to measure the activity of ENPP1 in vitro using *p*-Nph-5′-TMP as substrate, which produces a color change upon hydrolysis^22^. Despite the convenience and widespread use of this in vitro colorimetric assay^4^, we reasoned that ATP would be a more practical substrate for elucidating the kinetics of ENPP1 for the following reasons: (1) the specificity constant (*k*_cat_/*K*_M_) value of ATP is 6.74 folds higher than that of *p*-Nph-5′-TMP^22^, and (2) *p*-Nph-5′-TMP may also act as an allosteric modulator of ENPP1, which may be less susceptible to competitive inhibition^4^. Therefore, the measurement of ATP hydrolysis by monitoring its hydrolyzed product, inorganic pyrophosphate (PPi), would be a simpler and more accurate strategy for quantifying ENPP1 activity, without complications due to chemical derivatization and allosteric modulation. For this purpose, sensitive and real-time detection of PPi can be achieved by using Kyoto Green (xanthene dipicolylamine Zn(II) complex), a molecular fluorescent sensor extensively employed for the detection of PPi under physiological conditions and in synovial fluids^7,23–26^. However, to our knowledge, Kyoto Green has not been leveraged for screening of ENPP1 inhibitors. Here, we report the identification of ENPP1 inhibitors by employing Kyoto Green to measure their ability to inhibit ATP hydrolysis at physiological pH. Best inhibitors from the screening were then extensively characterized using a combination of Kyoto Green, commercial bioluminescence, and conventional colorimetric assays. Lastly, molecular docking and molecular dynamics (MD) simulations were performed to gain insights into structural factors that promote ENPP1 inhibition.

## Results and discussion

### Screening of potent inhibitors for ENPP1

**Figure 1:**
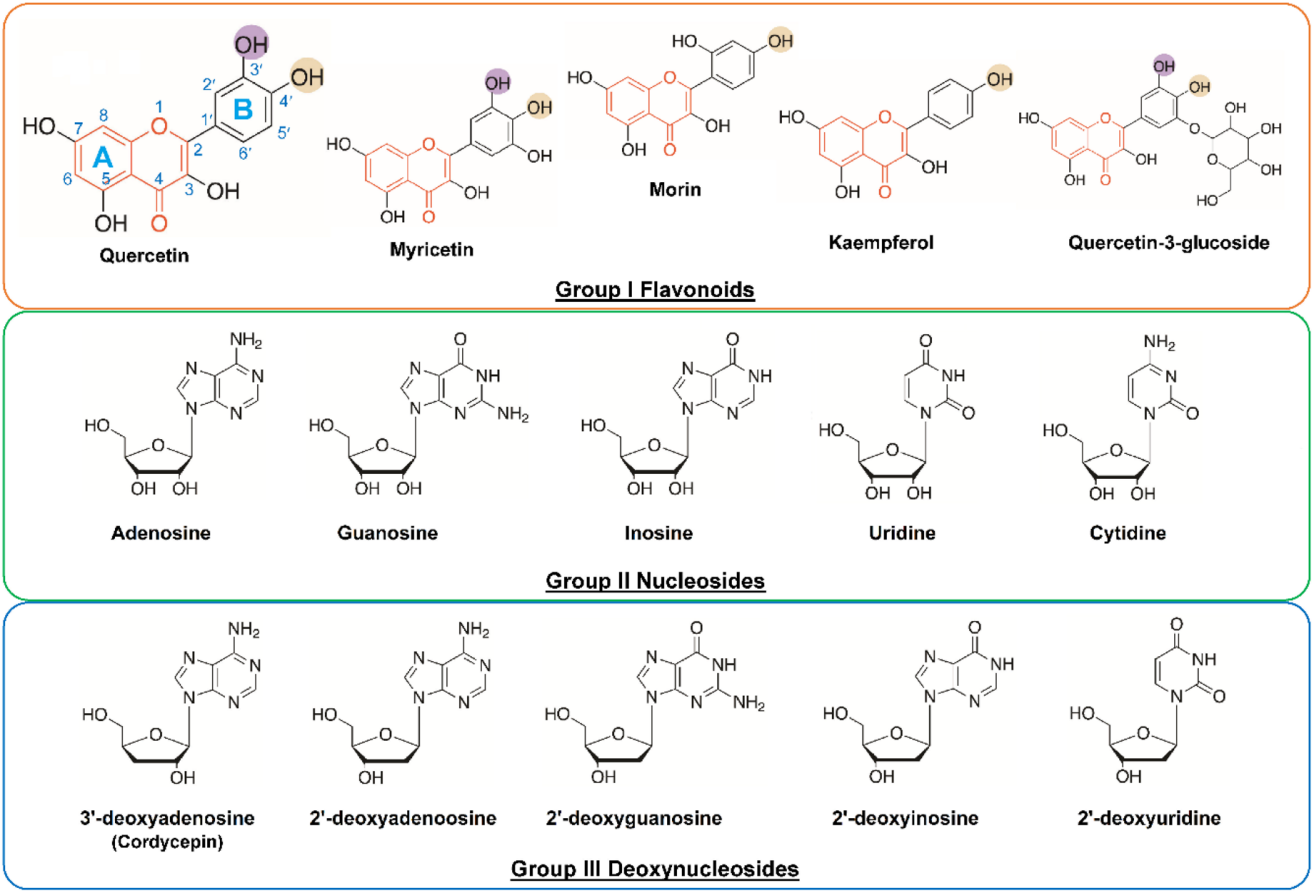
Structures of natural products tested in this study. Chromone scaffolds are highlighted in orange.

**Figure 2:**
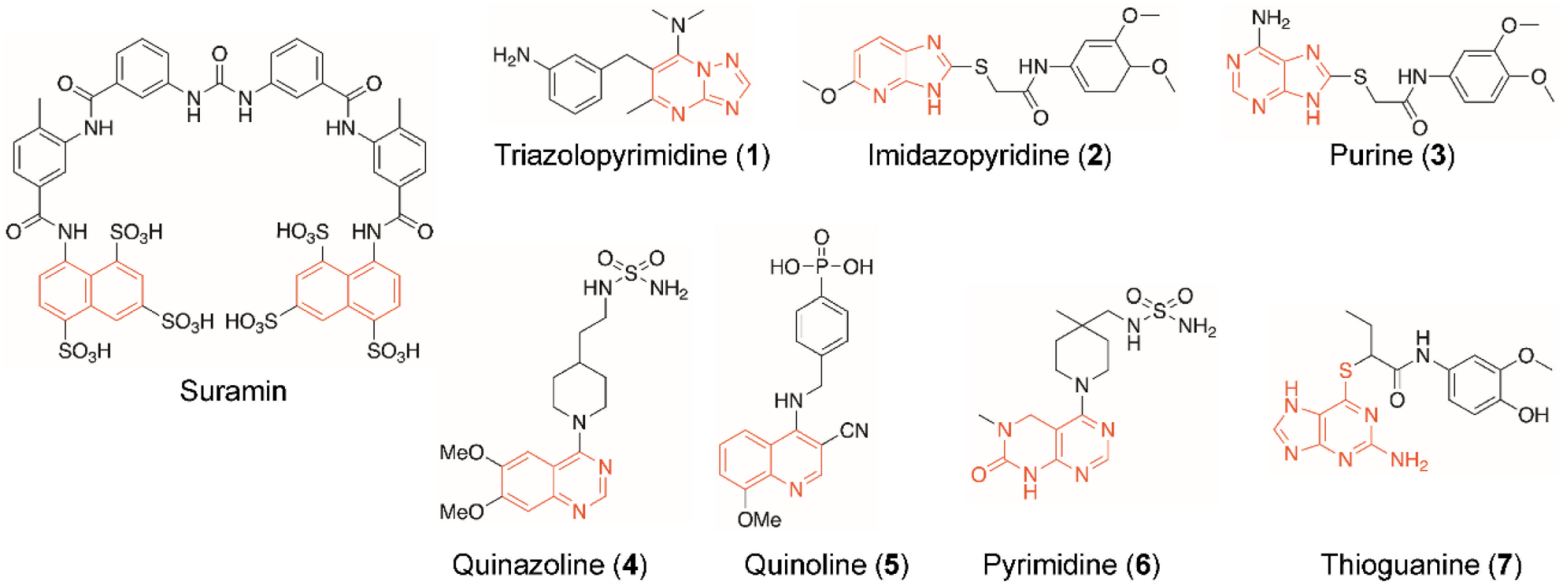
Molecular structures of notable ENPP1 inhibitors reported to date. Orange highlight denotes the basic scaffold of each structure: naphthalene; 1,2,4-triazolo[1,5-a]pyrimidine; 1H-imidazo[4,5-b]pyridine; purine; quinazoline; quinolone; (3,4-dihydropyrimido[2,3-d]pyrimidin-2(1H)-one; and thioguanine.

A total of 15 compounds derived from natural products were selected and classified into 3 groups as shown in Fig. 1. Group I includes 5 flavonoids with chromone scaffolds: quercetin, myricetin, kaempferol, morin, and quercetin glucoside. Group II consists of 5 nucleosides: adenosine, guanosine, uridine, cytidine, and inosine. Group III contains 5 deoxynucleosides including 2′-deoxyguanosine, 2′-deoxyuridine, 2′-deoxyinosine, 2′-deoxyadenosine, and 3′-deoxyadenosine (cordycepin). The flavonoids in Group I were chosen due to the presence a chromone ring, which structurally resembled a purine ring and may therefore inhibit ENPP1 in a manner similar to the previously reported purine-analogue inhibitors such as quinazoline and imidazopyridine^11,13,27–29^ (Fig. 2). Furthermore, three of the Group I members, quercetin, myricetin and kaempferol, were known inhibitors of xanthine oxidase^30^ and adenosine deaminase^31^, both of which use purine-containing substrates similar to ENPP1. Nucleosides (Group II) and deoxynucleosides (Group III) were included in the screening based on the report that AMP was a potent inhibitor ENPP1^27^. Notably, cordycepin, a member of Group III, is widely known for its numerous health benefits^32,33^.

**Figure 3:**
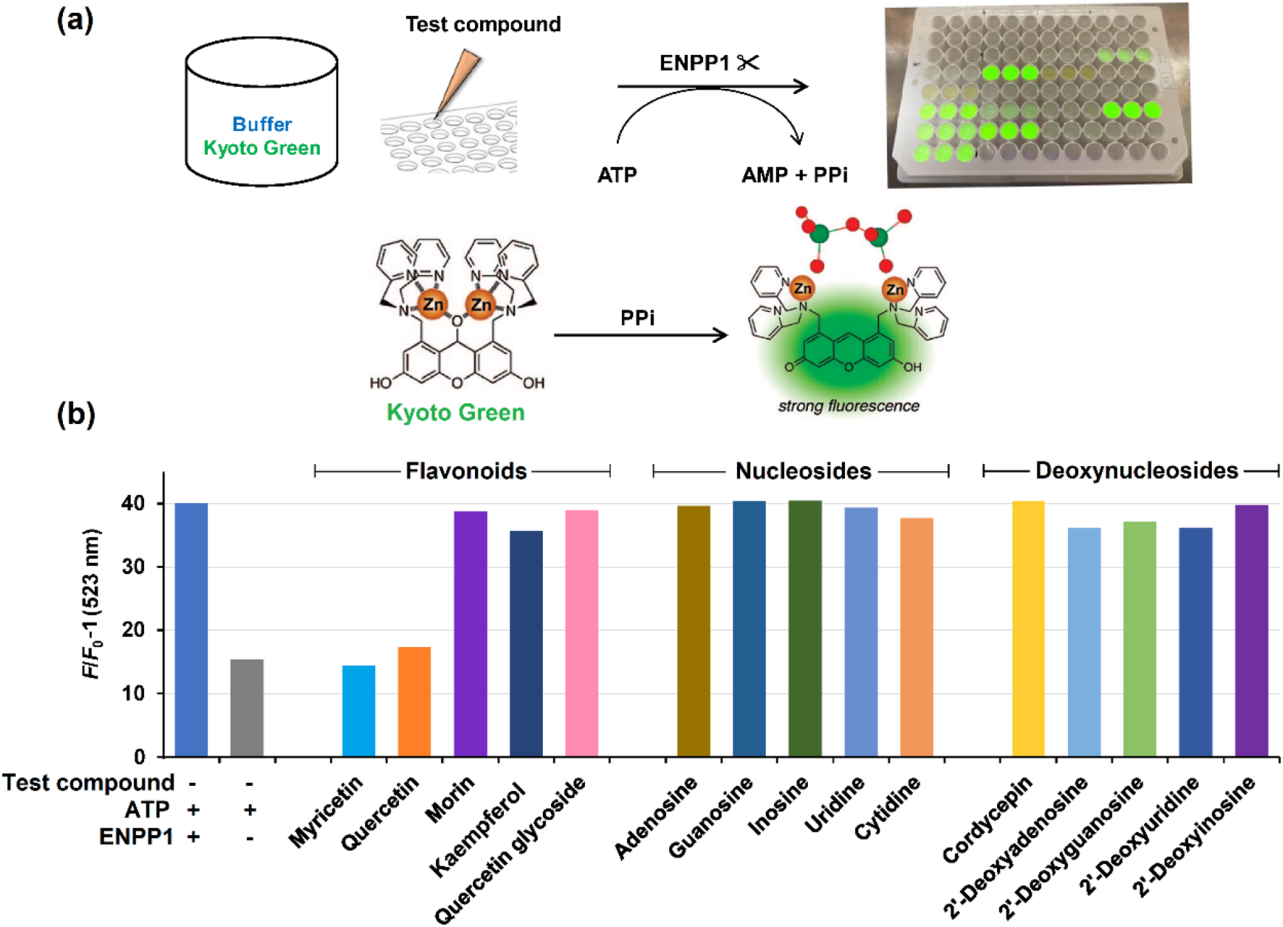
Kyoto Green-assisted screening of ENPP1 inhibitors. (**a**) Schematic diagram of the ENPP1 assay using Kyoto Green in a 96 well plate. (**b**) Fluorescence change (*F*/*F*_0_-1) of Kyoto Green after 20 min of ATP (2 μM) hydrolysis catalyzed by ENPP1 in the presence of each natural product (10 μM). For compounds with a solubility limit < 10 μM, the maximum soluble concentrations were used instead. Assay condition: 1 μM Kyoto Green in 50 mM HEPES buffer (pH 7.4) containing 10 mM NaCl, 1 mM MgCl_2_, 0.02 mM Zn(NO_3_)_2_, and 11.5 nM ENPP1. Excitation and emission wavelengths were 488 and 523 nm, respectively. *F*_0_ corresponds to fluorescence intensity of Kyoto Green in the buffer solution without ENPP1.

ATP was employed as a substrate for inhibitor screening in this study due to its role as a natural substrate of ENPP1 and the highest *k*_cat_/*K*_M_ (674 × 10^3^ M^−1^ s^−1^) among all the substrates^22^. The catalytic activity of ENPP1 was assessed by monitoring the amount of PPi generated from ATP hydrolysis over time, measured with the Kyoto Green fluorescent sensor as shown in Fig. 3a. This assay was conducted at a pH of 7.4 to mimic physiological conditions. To ensure the stability of Kyoto Green throughout the duration of the assay, the fluorescence emission profiles of Kyoto Green in the presence of ENPP1, ATP, AMP or PPi were monitored for 10 min (Supplementary Information Figure S1). The results show that the fluorescent emission of Kyoto Green at 523 nm was constant throughout the experiment, with the intensity resulting from PPi being approximately 3.8-fold higher than ATP. The stark difference in intensity between ATP and PPi was expected to provide a detection window when ATP is enzymatically hydrolyzed to PPi with high sensitivity and linearity. (Supplementary Information Figure S1 and S2). As shown in Fig. 3b, the solution containing ENPP1, ATP, and Kyoto Green displayed a significant increase in relative fluorescence change (*F*/*F*_0_ −1) after 20 min compared to the control without ENPP1. Strikingly, two of Group I inhibitors, myricetin and quercetin, reduced the fluorescence change by over 90% compared to the control group without an inhibitor. The remaining 13 candidates for inhibitor, however, did not significantly decrease fluorescence change. Considering that two of these ineffective inhibitors, morin and kaempferol, harbor a flavonoid ring similar to myricetin and quercetin, it could be inferred that the number and arrangement of hydroxyl groups on the B-ring may play a pivotal role in ENPP1 inhibition. Furthermore, we observed that quercetin-3-glucoside did not inhibit ENPP1, suggesting that the presence of a bulky sugar moiety may adversely affect ENPP1 inhibition. Finally, despite the previous report of AMP as an ENPP1 inhibitor, nucleosides (Group II) and deoxynucleosides (Group III) did not suppress the fluorescence change of Kyoto Green.

### Inhibition efficiency of quercetin and myricetin toward ENPP1

**Figure 4:**
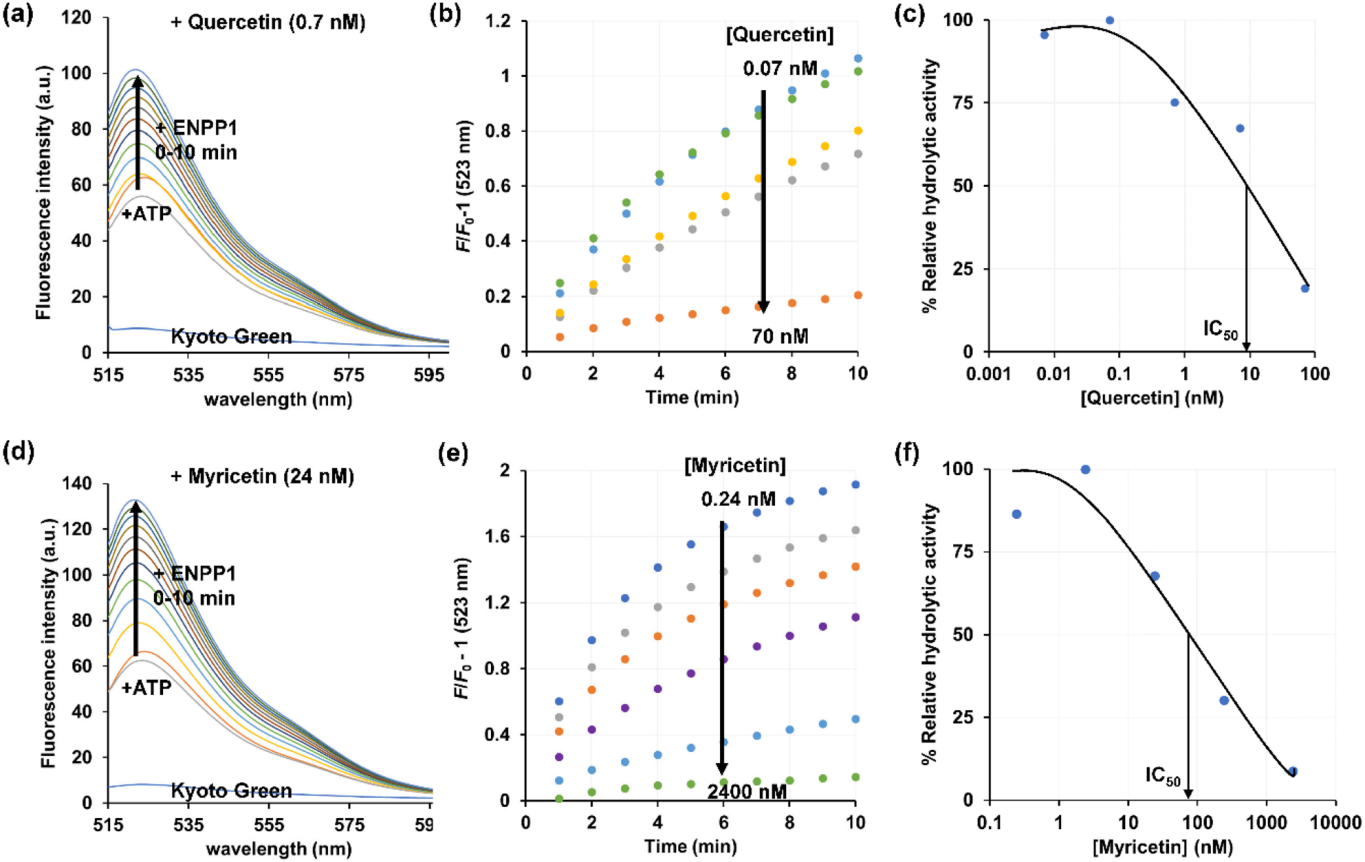
Inhibition efficiency of quercetin and myricetin. Time course of fluorescence intensity (**a**, **d**) and fluorescence change (*F*/*F*_0_-1) of Kyoto Green (**b**, **e**), and % relative hydrolytic activity of human ENPP1 in converting ATP to AMP and PPi at 5 min of reaction (**c**, **f**). The experiments were conducted in the presence of various concentrations of quercetin (**a**–**c**) and myricetin (**d**–**f**). *F*_0_ refers to the fluorescent intensity of Kyoto Green at 0 min of reaction. Assay conditions: 1 μM Kyoto Green, 1 μM ATP in 50 mM HEPES buffer (pH 7.4) containing 10 mM NaCl, 1 mM MgCl_2_, 0.02 mM Zn(NO_3_)_2_, and 3.2 nM ENPP1. Excitation and emission wavelengths were 488 and 523 nm, respectively.

Employing Kyoto Green to monitor ATP hydrolysis, we investigated the kinetics and efficiency of quercetin and myricetin in inhibition of ENPP1. The time course of ENPP1 catalysis in the presence of quercetin or myricetin revealed that the inhibitory effect increased as the inhibitor concentration increased (Supplementary Information Figure S3). To rule out the reduction in fluorescence change due to direct interaction between Kyoto Green and inhibitor, the assay was conducted with Kyoto Green, PPi, and various concentrations of quercetin (0.007–700 nM), which revealed that the fluorescence change did not depend on the concentration of quercetin at 95% confidence level (Supplementary Information Figure S4). By fitting the inhibition curve of quercetin (Fig. 4), the half maximal inhibitory concentration (IC_50_) of quercetin was determined to be 4.5 ± 0.9 nM. The value of *K*_i_ was estimated to be 4.0 ± 0.8 nM, using the Cheng–Prusoff equation^13^ with an estimated *K*_M_ value of 8170 nM^22^.

To benchmark the performance of the Kyoto Green method, the inhibitory effect of quercetin was re-evaluated using a commercial luciferase-based ATP assay kit (Supplementay Information Figure S5) and the conventional colorimetric method using *p*-Nph-5′-TMP. The commercial kit produced an IC_50_ value of 5.5 ± 0.6 nM and *K*_i_ value of 4.9 ± 0.5 nM (Table 1). The conventional colorimetric method yielded a *K*_i_ value of 159 ± 73 nM, which was approximately 40-fold higher than the values determined through the Kyoto Green and commercial ATP assays. The higher *K*_i_ observed, which suggested weaker inhibition, may be attributed to the fact that *p*-Nph-5′-TMP can act as an allosteric regulator of ENPP1^4^. This could potentially render the enzyme less susceptible to competitive inhibition by quercetin compared to when ATP was the substrate. Relative to myricetin and other known ENPP1 inhibitors shown in Fig. 2, quercetin exhibited higher inhibitory efficiency than myricetin (~ 8 folds), suramin (~ 188 folds), and compound **1**–**4** (Supplementary Information Table S1) using Kyoto Green fluorescence method. Notably, the *K*_i_ of quercetin was also comparable to compound **5**–**7**, which are among the most potent ENPP1 inhibitors reported to date (Fig. 2 and Supplementary Information Table S1).

**Table 1:** *K*_i_ values of quercetin, myricetin and suramin toward human soluble ENPP1.

Compounds	Substrates	Methods (Reporters)	pH	*K*_i_ (nM)	References
Quercetin	ATP	Fluorescence (Kyoto Green)	7.4	4.0 ± 0.8	This study
	ATP	Bioluminescence (Luciferin/luciferase)	7.4	4.9 ± 0.5	This study
	*p*-Nph-5′-TMP	Colorimetry (*p*-nitrophenol)	7.4	159 ± 73	This study
Myricetin	ATP	Fluorescence (Kyoto Green)	7.4	32 ± 2.2	This study
	ATP	Bioluminescence (Luciferin/luciferase)	7.4	51 ± 0.6	This study
	*p*-Nph-5′-TMP	Colorimetry (*p*-nitrophenol)	7.4	116 ± 24	This study
	*p*-Nph-5′-TMP	Colorimetry (*p*-nitrophenol)	7.4	(432)*	16
	*p*-Nph-5′-TMP	Colorimetry (*p*-nitrophenol)	9.5	(414)*	
Suramin	ATP	Fluorescence (Kyoto Green)	7.4	753 ± 55	This study
	ATP	Capillary Electrophoresis	9.0	780	4
	*p*-Nph-5′-TMP	Colorimetry (*p*-nitrophenol)	7.4	260 ± 25	This study
	*p*-Nph-5′-TMP	Colorimetry (*p*-nitrophenol)	9.0	1070	4

*IC_50_ value is shown when *K*_i_ is not available from the original study.

It is worth noting that, in addition to its inhibitory effect at pH 7.4, quercetin was also capable of inhibiting ENPP1 at pH 9.0, but this effect was observed only for the first 10 min of the time course (Supplementary Information Figure S6). The lack of inhibitory effect at later time points could be due to the rapid oxidation of quercetin in alkaline solutions^34^. Conversely, quercetin was observed to be highly stable in 50 mM HEPES buffer (pH 7.4) for 60 min (Supplementary Information Figure S7).

Evaluating the inhibitory effect of myricetin with Kyoto Green method, the commercial ATP detection kit and the colorimetric *p*-Nph-5′-TMP assay resulted in IC_50_ values of 35 ± 2.5, 58 ± 0.6 and 158 ± 31 nM, respectively (Fig. 4). The colorimetric method generated a lower IC_50_ value than previously reported (432 nM)^16^, possibly because a different buffer was used in this study (Table 1). Similar to quercetin, the inhibition efficiency of myricetin depended on the type of substrate, with a lower *K*_i_ (32 ± 2 nM, Kyoto Green method) for ATP than *p*-Nph-5′-TMP (116 ± 24 nM). The strong inhibitory effect observed for myrecitin was not caused by direct interaction between the inhibitor and Kyoto Green, as the sensitivity of Kyoto Green was independent of myricetin concentration (Supplementay Information Figure S8). Finally, suramin, one of the known ENPP1 inhibitors shown in Fig. 2, produced an IC_50_ value of 845 ± 61 nM (*K*_i_ = 753 ± 55 nM) in the Kyoto Green assay, which was comparable to the reported value^4^ (Table 1). The sensitivity of Kyoto Green was also not affected by high concentrations of suramin (Supplementay Information Figure S9). Taken together, the results strongly highlight the potential of Kyoto Green as an accurate, robust, and facile tool for screening ENPP1 inhibitors in the presence of the natural substrate ATP.

### Molecular docking

Docking simulations were performed to investigate the binding mode of each flavonoid with human ENPP1, utilizing the AutoDock Vina and DockThor programs. To validate the accuracy of our docking protocol, we conducted a redocking experiment with the co-crystallized quinazoline **4** to predict its position within the enzyme binding pocket, and compare the predicted position to that in the crystal. The superposition between poses obtained from redocking and crystallography revealed that the top prediction for inhibitor **4** closely matched the crystallographic pose (root-mean-square deviation, RMSD values of 0.57 Å for AutoDock Vina and 1.05 Å for DockThor, as shown in Supplementary Information Figure S10). Having confirmed that the docking protocol utilizing both programs was reliable, we proceeded to dock all the compounds of interest against human ENPP1, with compound **4** and AMP serving as controls (Supplementary Information Table S2.) The docking results suggested that all the flavonoids (myricetin, quercetin, kaempferol, morin, and quercetin-3-glucoside) exhibited higher binding affinity than AMP, albeit with slightly lower binding energy than inhibitor **4**. These results contradict the findings from our experiments described earlier, in which only quercetin and myricetin were effective in inhibiting human ENPP1 (Fig. 3b and Table 1). As these experiments were performed with aqueous solutions, it is plausible that the molecular docking simulations performed in a gas phase may not have accurately represented the true dynamics of ligand binding in the aqueous environment.

**Figure 5:**
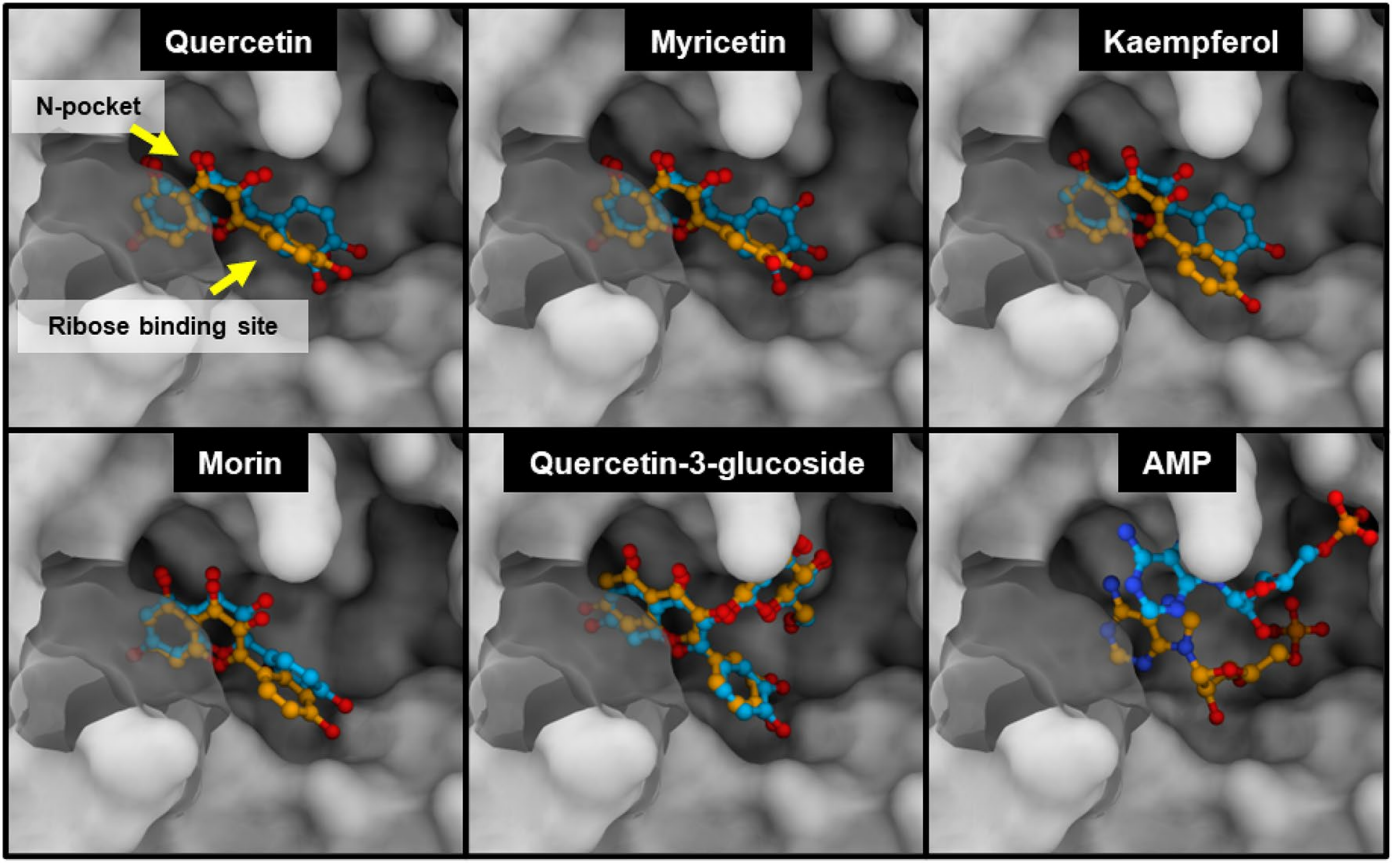
Docked conformations of quercetin, myricetin, kaempferol, morin, quercetin-3-glucoside and AMP bound to the human ENPP1’s active site. These models were generated using AutoDock Vina (orange) and DockThor (light blue) programs. The active site contains two binding pockets: N-pocket and ribose binding site.

To assess the agreement between the two programs used for molecular docking, we overlaid the structures of docked compounds obtained from both programs, which shows that both docking tools produced similar orientations for all of the flavonoids (Fig. 5). Specifically, the chromone ring of each flavonoid was effectively inserted into the nucleotide-binding pocket (N-pocket) of human ENPP1, formed by residues F257, R295, W322, P323, D326, and Y340. These residues were equivalent to F239, R277, W304, P305, D308 and Y322 in mouse ENPP1^35^. The preferential binding of the chromone ring to the N-pocket was similar to the observed behavior of the previously reported inhibitor **4**, in which the quinazoline core deeply projected into the N-pocket and formed interactions with F321, P323, D326, and Y371^29^. These results indicate that the N-pocket of human ENPP1 may be highly specific to fused bicyclic rings with high planarity, such as the adenine moiety of adenine nucleotide substrates.

Furthermore, the docking results revealed that ring B of each compound was positioned at the reported binding sites of ribose in AMP and the piperidine ring in inhibitor **4**^29^. These findings are consistent with the binding mode of myricetin with human ENPP1 determined in a recent docking study using Glide software^16^. Nevertheless, it should be noted that the conformations of AMP predicted by AutoDock Vina and DockThor in this study were significantly different. In the case of AutoDock Vina, the adenine and ribose moieties of AMP were predicted occupy the N-pocket and the ribose binding site, respectively, with the phosphate group pointing toward the zinc ions in the active site. This conformation closely resembled that observed in the human ENPP1–AMP structure (PDB entry 6WFJ^29^, Supplementary Information Figure S11). In contrast, DockThor placed the AMP molecule at a more solvent-exposed region within the active site, which differed from its location in the X-ray structure (Supplemenatry Information Figure S11). The different orientations predicted by both programs could be attributed by DocThor’s incorporation of electrostatic and solvation terms^36^, a feature absent in AutoDock Vina’s scoring function^37^. Nonetheless, in this study, the docking results from AutoDock Vina were considered more reliable because it successfully replicated the crystallographic poses of AMP and inhibitor **4**. Therefore, the structures obtained through AutoDock Vina were used for subsequent MD simulations.

### Molecular dynamics: system stability and flexibility

**Figure 6:**
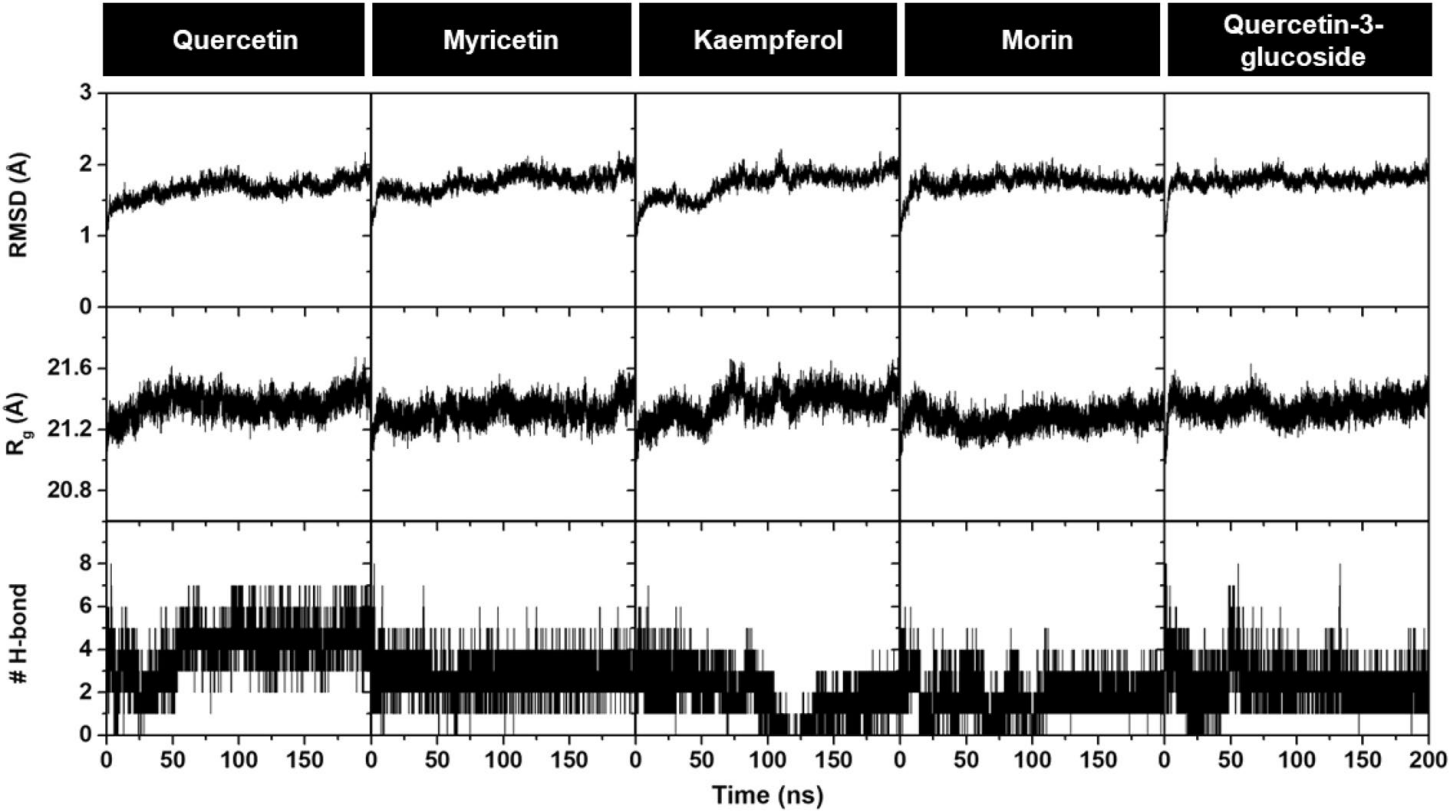
Time evolution of (top) RMSD, (middle) R_g_ and (bottom) # H-bond for flavonoid-human ENPP1 complexes during 200 ns MD simulations.

To accurately capture the dynamic behavior of protein–ligand complexes in an aqueous solution, 200-ns MD simulations of the human ENPP1–flavonoid complexes were conducted. The structural stability and flexibility of the complexes were assessed from various parameters, including RMSD, the radius of gyration (R_g_), the number of intermolecular H-bonds (# H-bonds), and root-mean-square fluctuation (RMSF) (Fig. 6 and Supplementary Information Figure S12). Given the substantial size of the simulated system (> 800 amino acids), our analysis primarily focused on the RMSD profiles of protein atoms within the catalytic domain of ENPP1 (390 residues) and the heavy atoms of the ligand. Additionally, the R_g_ plots for C_α_ atoms within the catalytic domain were monitored to further confirm the equilibration of the systems and explore the compactness of the protein structure. The results show that all of systems reached the equilibrium after ~ 100 ns and maintained a relatively consistent fluctuation of ~ 1.7–1.8 Å until the end of the simulation period. The R_g_ values, averaged from the last 50 ns of simulation for all systems, were ~ 21.3–21.5 Å, indicating that the overall protein structure remained relatively compact upon ligand binding. We also investigated # H-bonds formed between the protein and the ligand throughout the simulation. The # H-bonds in these complexes exhibited fluctuations during the first 50–100 ns before becoming stabilized for the rest of the simulation. However, for kaempferol, the fluctuation continued until ~ 130 ns. Moreover, it was observed that during the period from 150 to 200 ns, all the flavonoids consistently formed 2–3 H-bonds with the binding site residues, except for quercetin, which formed 4–5 H-bonds (Fig. 6). In summary, the relatively small fluctuations in RMSD, R_g_, and # H-bonds confirmed the stability of the dynamic equilibria in all simulated systems.

Furthermore, for each system, we calculated and compared the RMSF (average deviation of the C_α_ atoms of amino acid residues) values of the ligand-bound and apo enzyme to estimate the degree of amino acid mobility in response to ligand binding (Supplementary Information Figure S12). In general, the bound state exhibited fluctuations in RMSF profiles similar to the apo state, with the fluctuations being most pronounced within the catalytic domain (residues 208–597). Certain residues of the bound state also displayed low flexibility similar to their counterparts in the apo form, including residues at the N-pocket (F257, R295, W322, P323, D326, and Y340), the ribose binding site (L290, Y371, and E373), and the catalytic center (D218, T256, N277, D376, H380, D423, H424, and H535). Altogether, this information suggested that the protein conformation of the catalytic domain, especially the active site residues, remained stable when flavonoids bound to the enzyme.

### Molecular dynamics: binding free energy of protein–ligand complexes

The binding strength of each flavonoid to human ENPP1 was estimated based on the binding free energy ($\Delta {G}_{\text{bind}}$) computed using the Molecular Mechanics Generalized Born Surface Area (MM/GBSA) approach^38^. The $\Delta {G}_{\text{bind}}$ and energetic components of each complex, averaged over 500 snapshots from the last 50 ns, are provided in Table 2. For the ENPP1–quercetin complex, the calculated interaction energies ($\Delta {E}_{\text{MM}}$) revealed that the electrostatic interaction ($\Delta {E}_{\text{ele}}$) was stronger than the van der Waals interaction ($\Delta {E}_{\text{vdW}}$), consistent with the higher # H-bonds detected in this system (Fig. 6). In contrast, for myricetin and kaempferol systems, high $\Delta {E}_{\text{vdW}}$ suggested that the van der Waals interaction was the dominant driving force in stabilizing the complex. In the case of the morin- and quercetin-3-glucoside-ENPP1 complexes, both $\Delta {E}_{\text{vdW}}$ and $\Delta {E}_{\text{ele}}$ contributed almost equally to ligand binding, with an energy contribution of approximately –28 kcal/mol. However, the negative value of $\Delta {E}_{\text{vdW}}+\Delta {G}_{\text{sol}}^{\text{nonpolar}}$ indicated that nonpolar interactions predominantly contributed to the stabilization of the binding between human ENPP1 and all of the flavonoids, as opposed to the unfavorable contribution (positive value) of the polar terms ($\Delta {E}_{\text{ele}}+\Delta {G}_{\text{sol}}^{\text{ele}}$).

**Table 2:** Binding free energy components (kcal/mol) for the complexation of each flavonoid with human ENPP1, as determined using the MM/GBSA method.

	Quercetin	Myricetin	Kaempferol	Morin	Quercetin-3-glucoside
$\Delta {\text{E}}_{{{\text{vdW}}}}$	–24.90 ± 0.13	–29.41 ± 0.11	–25.71 ± 0.11	–28.39 ± 0.10	–28.09 ± 0.13
$\Delta {\text{E}}_{{{\text{ele}}}}$	–82.02 ± 0.44	–9.83 ± 0.07	–14.20 ± 0.23	–28.36 ± 0.53	–27.21 ± 0.41
$\Delta {\text{E}}_{{{\text{MM}}}}$	–106.92 ± 0.41	–39.24 ± 0.11	–39.91 ± 0.25	–56.75 ± 0.51	–55.29 ± 0.38
$- {\text{T}}\Delta {\text{S}}$	13.98 ± 0.63	14.58 ± 0.53	15.65 ± 0.69	15.71 ± 0.55	17.36 ± 0.67
$\Delta {\text{G}}_{{{\text{sol}}}}^{{{\text{ele}}}}$	85.08 ± 0.38	15.85 ± 0.08	25.22 ± 0.19	39.58 ± 0.49	43.31 ± 0.37
$\Delta {\text{G}}_{{{\text{sol}}}}^{{{\text{nonpolar}}}}$	–4.35 ± 0.01	–3.21 ± 0.01	–3.63 ± 0.01	–3.64 ± 0.01	–4.53 ± 0.01
$\Delta {\text{G}}_{{{\text{sol}}}}$	80.73 ± 0.38	12.64 ± 0.08	21.59 ± 0.19	35.94 ± 0.49	38.78 ± 0.37
$\Delta {\text{G}}_{{{\text{total}}}}$	–26.19 ± 0.13	–26.60 ± 0.10	–18.31 ± 0.13	–20.81 ± 0.10	–16.51 ± 0.10
$\Delta {\text{G}}_{{{\text{bind}}}}$	–12.21 ± 0.64	–12.02 ± 0.54	–2.66 ± 0.70	–5.10 ± 0.56	0.85 ± 0.68

Data are shown as mean ± standard error of the mean (SEM).

**Figure 7:**
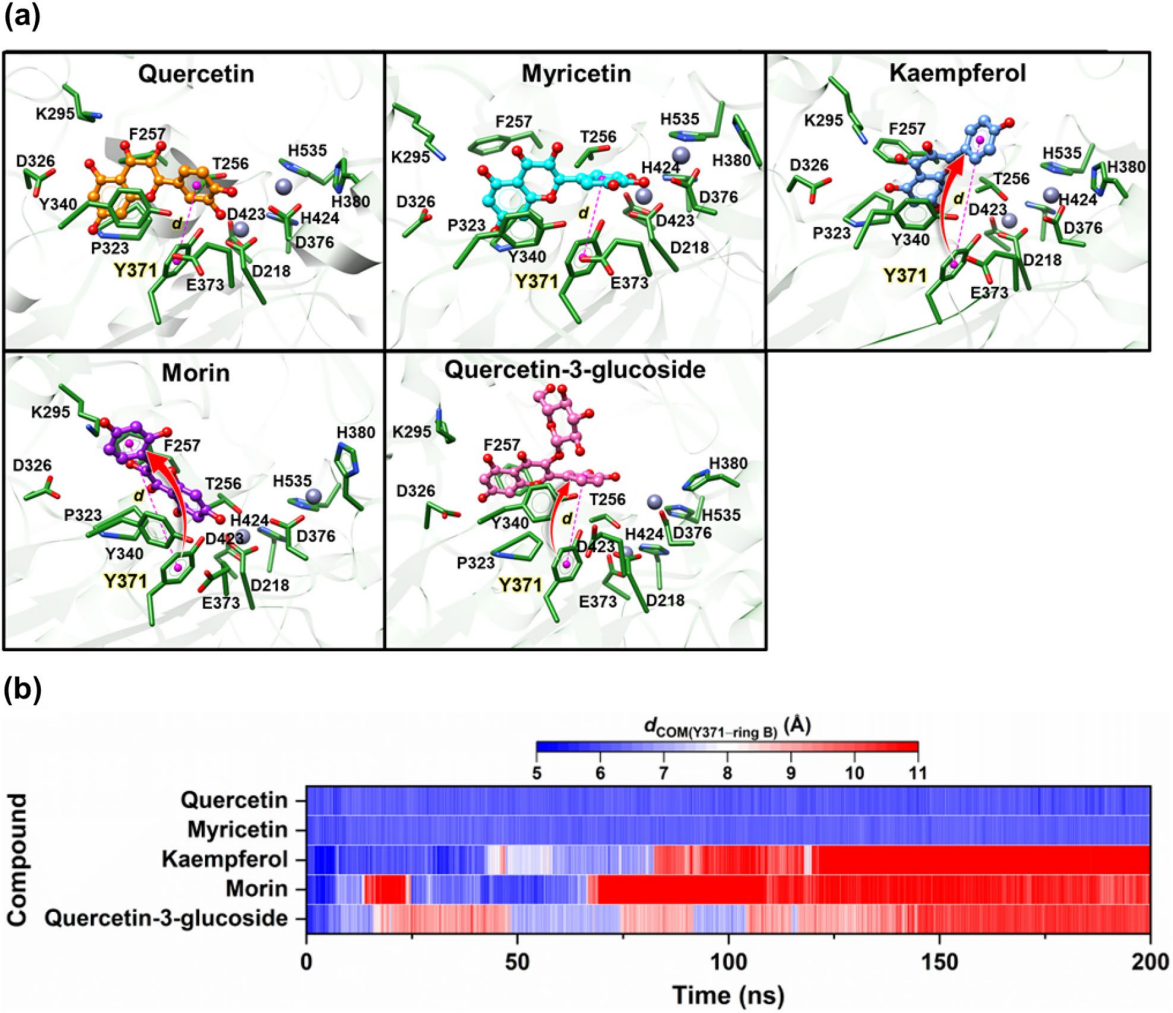
(**a**) Representative 3D structures showing the orientation of each compound in the human ENPP1 active site, drawn using the final MD structures. Red arrow represents the trajectory of the B ring as it moves out of the ribose binding site. Pink dashed line indicates the distance between the center of mass of the Y371 phenyl ring and B ring. (**b**) Time evolution of *d*_COM(Y371–ring B)_ for quercetin, myricetin, kaempferol, morin and quercetin-3-glucoside, bound to human ENPP1 during 200 ns MD simulations. The color scale from blue to red represents the increasing distances from 5 to 11 Å.

The highly negative $\Delta {G}_{\text{bind}}$ values of quercetin and myricetin (–12.21 and –12.02 kcal/mol, respectively) indicated a stronger binding affinity of these inhibitors to human ENPP1 compared to kaempferol, morin, and quercetin-3-glucoside (Table 2). These findings were in line the relative inhibition efficiency observed in the enzymatic assays, where quercetin and myricetin were deemed the most effective inhibitors of Group I (Fig. 3 b, Table 1). The superior affinity of quercetin and myricetin may be attributed to their lower mobility within the enzyme binding pocket, resulting in a tighter binding to the surrounding residues of human ENPP1 (Fig. 7).

It is worth mentioning that the values of $\Delta {G}_{\text{bind}}$ computed using this end-state method does not represent the absolute binding free energy of protein–ligand complexes. Nonetheless, it offers a more cost-effective solution than pathway approaches (e.g., free energy perturbation and thermodynamic integration) and provides greater accuracy than docking scoring functions^39^. Alternatively, a computationally inexpensive tool called τ-random acceleration molecular dynamics (τRAMD) may be employed to calculate the relative drug-target residence time of each complex compound. This information can be used to complement the end-point methods in the ranking of compounds and gaining insights into their protein–ligand dissociation mechanisms^40^.

To further investigate the movement of ligands within the enzyme binding pocket, which could influence binding affinity, we examined the representative MD snapshots of all the flavonoid-human ENPP1 complexes. As shown in Fig. 7a, the MD structure of each complex, with an exception of morin, indicated a stable binding of the chromone ring within the N-pocket of human ENPP1. In contrast, the B ring of kaempferol, morin and quercetin-3-glucoside could not maintain their binding at the ribose binding site, potentially reducing their ability to exclude nucleotide substrates from the active site. This finding may explain the observed poor inhibitory activity of these three compounds in the earlier enzymatic assays.

To further corroborate our visual interpretation of the MD snapshots, we monitored the time evolution of the center-of-mass distance between the B ring of each flavonoid and the phenyl ring of Y371, a prominent amino acid at the ribose binding site (*d*_COM(Y371–ring B)_) (Fig. 7b). The results show that the B ring of quercetin and myricetin was indeed positioned near Y371, exhibiting relatively stable *d*_COM(Y371–ring B)_ values of ~ 5–6 Å over the course of the MD simulation. Conversely, the *d*_COM(Y371–ring B)_ values of the remaining complexes exceeded 8 Å at various time points during the simulation, indicating that the B ring could not maintain its binding. As both kaemperfol and morin lack a 3′-OH group, it is conceivable the presence of -OH at 3′ and/or 4′ positions is required to form a hydrogen bond (H-bond) with the active site residues. The resulting H-bonding then helps orient the scaffold for optimal binding within the ribose binding site of human ENPP1. To test this hypothesis, we tracked H-bond occupations between human ENPP1 residues and -OH groups on the B ring of quercetin and myricetin over the last 50 ns. The results show that the 3′-OH of quercetin and myricetin engaged in a strong H-bond with the η-hydroxyl group of Y371 and the γ-carboxylate group of D376 at 91.7% and 98.4%, respectively (Supplementary Information Figure S13), leading to the stabilization of the B ring within the ribose binding site. Although quercetin-3-glucoside possesses a 3′-OH group, its bulky glucoside group may sterically clash with the ligand binding site, thereby causing the compound to move outward to the solvent-exposed area.

## Materials and methods

### Materials

Kyoto Green was synthesized following the procedure described in Ojida et al.^23^. Magnesium chloride (MgCl_2_), zinc nitrate (Zn(NO_3_)_2_), HEPES minimum 99.5% titration (C_8_H_18_N_2_O_4_S), *p*-nitrophynyl thymidine 5′-monophosphate (*p*Nph-5′-TMP), sodium pyrophosphate decahydrate (NaPPi), adenosine 5′-triphosphate disodium salt (ATP), and adenosine 5′-monophosphate disodium salt (AMP) were products of Sigma-Aldrich (St. Louis, MO, USA) or Merck (Darmstadt, Germany). Cordycepin, uridine, cytidine, inosine, thymidine, kaempferol, 2′-deoxyadenosine, 2′-deoxyinosine, 2′-deoxyuridine, and 2′-deoxyguanosine were purchased from Fujifilm Wako Pure Chemical Corporation (Japan). Myricetin, quercetin dihydrate and guanosine were commercially available from Alfa Aesar (UK). Recombinant human ENPP1 was purchased from R&D systems (Minneapolis, USA).

### Fluorescent screening of ENPP1 inhibitors using ATP as a substrate and Kyoto Green as a reporter

The screening was performed with a total volume of 200 µl on a white 96-well plate, using the condition described by Yongwattana et al.^7^. The assay solution contained 50 mM HEPES buffer (pH 7.4), 10 mM NaCl, 1 mM MgCl_2_, 20 µM Zn(NO_3_)_2_, 2 µM ATP, 1 µM Kyoto Green and each test compound at 10 µM or maximum soluble concentration in water. Background fluorescence of the buffer containing Kyoto Green was measured by a microplate reader (Varioskan Flash, Thermo Fisher Scientific, Massachusetts) and denoted as *F*_0_. The enzymatic reaction was initiated by adding 11.5 nM ENPP1, after which the fluorescent emission (*F*) was measured using the kinetic mode at intervals of 30 s for 30 min (emission and excitation wavelengths of 523 and 488 nm, respectively). To make graphical presentation more intuitive to readers, the fluorescence response is expressed as fluorescence change (*F*/*F*_0_-1) because its value is zero at t = 0, while *F*/*F*_0_ would have a value of 1 at the starting point.

### Fluorescent determination of IC_50_ using Kyoto Green

For the concentration-dependent inhibition test, myricetin and suramin solutions were prepared by directly dissolving the compounds in water, whereas quercetin was dissolved in absolute ethanol due to its poor solubility in water. To initiate the enzymatic assay, each solution in the dilution series was mixed with Kyoto Green (1 μM) in the assay solution (1 μM ATP in 50 mM HEPES buffer (pH 7.4) containing 10 mM NaCl, 1 mM MgCl_2_, 0.02 mM Zn(NO_3_)_2_) before adding ENPP1 (3.2 nM). The conversion of ATP to AMP and PPi was monitored by real-time measurements of fluorescence change (*F*/*F*_0_-1) using a spectrofluorometer (Jasco FP6500, Japan). The initial velocity was calculated from the data obtained during the first 5 min of reaction (n = 3). Then, IC_50_ was calculated from the inhibition curve constructed using the initial velocity data obtained at various concentration of the test compound. *K*_i_ was calculated from the IC_50_ value following the procedure described in Carozza et al.^13^, using the Cheng-Prusoff equation: *K*_i_ = IC_50_ / 1 + [S] / *K*_M_^13^. *K*_M_ was set as 8.17 µM for human ENPP1, which was a previously reported value determined using ATP as a substrate at pH 9.0^22^.

To evaluate the potential interference of the test compounds with the sensitivity of Kyoto Green, each test compound was mixed with 1 µM PPi and 1 µM Kyoto Green in 10 mM NaCl, 1 mM MgCl_2_, 20 µM Zn(NO_3_)_2_ (pH 7.4), in the absence of ENPP1. The purpose of including 1 µM PPi in this solution was to mimic the condition where 1 μM ATP substrate has been completely converted to PPi (i.e., 100% hydrolytic reaction.) A negative control containing no PPi was also prepared. Then, the fluorescence emission was measured in the presence (referred to as *F*) and absence (referred to as *F*_0_) of PPi (emission and excitation wavelengths of 523 and 488 nm, respectively).

### Colorimetric UV–visible spectroscopic assay of ENPP1 using p-Nph-5′-TMP as a chromogenic substrate

A working solution (700 µl) was prepared from the following components: 10 mM NaCl, 1 mM MgCl_2_, 20 µM Zn(NO_3_)_2_, 100 µM *p*-Nph-5′-TMP in 50 mM HEPES buffer (pH 7.5). ENPP1 was introduced a final concentration of 6.5 nM to initiate the hydrolysis of *p*-Nph-5′-TMP, which generates a yellow product (*p*-nitrophenolate) exhibiting a strong absorbance at 397 nm. The absorbance at 397 nm was monitored using a UV–visible spectrometer (Jasco V-730, Japan) and the data were used to establish a time course. To determine of inhibitory efficiency of quercetin or myricetin, the experiment was repeated in the presence of each compound at different concentrations. The initial velocity was determined at different concentrations of the test compound, as described in the previous section. The initial velocity values were averaged and used to calculate relative hydrolytic activity, and IC_50_ was determined from the plot between relative hydrolytic activity and concentration. *K*_i_ was calculated from IC_50_ following the steps described in the preceding section, with *K*_M_ set at 222 µM based on a previous study that used *p*-Nph-5′-TMP a substrate at pH 9.0^22^. All experiments described in this section were performed in triplicate.

### Bioluminescent assay of ENPP1 using luciferin/luciferase assay kit

A tenfold dilution series of the test compound was mixed with human ENPP1 (3.2 nM) and ATP (1 µM) in 50 mM HEPES buffer (pH 7.5) containing 10 mM NaCl, 1 mM MgCl_2_, and 20 µM Zn(NO_3_)_2_ in a total volume of 200 µl. The solution was incubated at incubation at room temperature incubated at room temperature for 10 min and then heat-inactivated at 95 °C for 5 min. To evaluate the catalysis by ENPP1, a recombinant luciferase/luciferin (rL/L) test kit (ENLITEN® ATP assay system, Promega, Madison Wisconsin, USA) was employed to quantify ATP as the reaction progressed. Briefly, 100 µl of the reaction established above and 100 µl of the rL/L reagent from the kit were incubated in the dark for 3 min. Bioluminescence generated by the luciferin/luciferase reaction was measured using a microplate reader (Varioskan Flash, Thermo Fisher Scientific, Massachusetts) in the normal mode. All experiments were performed in triplicate. IC_50_ was calculated by plotting the ENPP1 hydrolytic activity derived from an average initial velocity against the concentration of test compound. The *K*_i_ value was calculated from the IC_50_ value using the Cheng-Prusoff equation as described in the preceding section, with the *K*_M_ of human ENPP1 set to 8.17 µM (a previously reported value obtained using ATP as a substrate and at pH 9.0)^22^.

### Statistical analysis

Three repeated measurements were performed for each experiment. Each data point is expressed as mean ± standard deviation. One-way analysis of variance (ANOVA) and Tukey’s test were used to establish the significance of differences (*p*-value < 0.05). All of the statistical analyses were performed using SPSS version 14.0 for Windows (SPSS Inc., Chicago, IL, USA).

## Computational studies

### System preparation and molecular docking

The X-ray structure of human ENPP1 bound to a known inhibitor (compound **4**) was retrieved from the Protein Data Bank (PDB code: 6WEV^29^). The 3D structures of all compounds were built utilizing GaussView 5.0, and subsequently fully optimized at the B3LYP/6-31G(d) level using Gaussian09 software (Gaussian, Inc., Wallingford, CT, USA)^41^. The SWISS-MODEL server^42,43^ was employed to model all missing residues in the human ENPP1 structure. The centroid of co-crystalized ligand in the crystal structure was designated as the site for molecular docking analysis using Autodock Vina^37^ with a rigid docking protocol. The software’s default parameters for cubic grid dimension were used (20 × 20 × 20 Å with x, y, and z coordinates of 35.1, –5.2, and 7.2, respectively). In addition, a molecular docking study was conducted using the DockThor-VS web server^37^. The docking grid was centered on the enzyme’s active site, with the grid size and coordinates (x, y, and z) identical to AutoDock Vina’s settings. The DMRTS genetic algorithm was configured using the standard parameters of the search algorithm, with the soft docking mode activated.

The binding mode of each compound with human ENPP1 was visualized using the UCSF Chimera^44^ and ChimeraX programs^45^. Docked complexes with the highest binding affinity (i.e., the first pose with the lowest Autodock Vina score) were selected as the starting points for molecular dynamics (MD) simulation. Prior to performing an MD simulation, the electrostatic potential (ESP) charges of the optimized ligands were computed at the HF/6-31G(d) level, then converted to restrained ESP (RESP) charges via the antechamber module of AMBER20^46^. The missing molecular parameters of the ligands were derived based on the general AMBER force field 2 (GAFF2)^47^ using the parmchk2 module, while the bonded and non-bonded parameters of the protein were refined with the AMBER ff19SB force field^48^. Hydrogen atoms missing from the protein–ligand complex were added using the LEaP module. Furthermore, each complex was solvated using the OPC explicit solvation model^49^ with a minimum distance of 10 Å between the solvation box edge and protein surface. Chloride counterions were randomly added to neutralize the overall charge of the system. During the minimization step, the introduced hydrogen atoms and solvent molecules were energetically minimized using 3000 steps of the steepest descent (SD) algorithm, followed by 1000 steps of the conjugated gradient (CG) algorithm.

### Molecular dynamics simulation

The structures of protein–ligand complexes obtained from the docking simulation were subjected to MD simulation for 200 ns under the periodic boundary condition with the isothermal–isobaric (*NPT*) scheme, using AMBER20 software package. The Particle Mesh Ewald (PME) summation method^50^ and a 10-Å cutoff distance were employed to model long-range electrostatic interactions and short-range nonbonded interactions, respectively. An integration time step of 2 fs was used in combination with the SHAKE algorithm^51^ to maintain the length of bonds involving hydrogen atoms. The target pressure (1 atm) of each simulated system was controlled by the Berendsen barostat^52^ with a pressure-relaxation time of 1 ps, while the temperature (300 K) was controlled by the Langevin thermostat^53^ with a damping frequency of 2 ps^−1^. The thermalization, equilibration, and production phases of individual MD simulations were established using the same protocol according to our previous study^54^. The MD trajectories were saved every 10 ps. For structural analysis, RMSD, R_g_, # H-bonds, RMSF, and *d*_COM(Y371–ring B)_ were calculated using the CPPTRAJ utility^55^ of AmberTools21. It is important to note that H-bonds were identified based on the following geometric criteria: (1) a hydrogen donor (HD)–acceptor (HA) distance of ≤ 3.5 Å and (2) a HA···H–HD angle of ≥ 120°. Meanwhile, the values of $\Delta {G}_{\text{bind}}$ were computed based on the MM/GBSA method using the MMPBSA.py module^56^ implemented in AMBER 20. To reduce computational resources, the change in solute entropy ($T\Delta S$) was estimated by a modified version of normal mode analysis (NMA) (referred to as truncated NMA). This approach was based on the 9 Å-truncated MD structures, as described by Sun et al.^57^.

## Conclusions

Using the combination of Kyoto Green, commercial bioluminescence kit, and the conventional colorimetric assay, we demonstrated that quercetin and myricetin were potent inhibitors of ENPP1, while morin, kaempferol and quercetin glucoside were ineffective as inhibitors. Computational analyses revealed that the chromone ring of each flavonoid tested in this study projected into the N-pocket of ENPP1. In silico analysis of H-bond occupations suggested that the presence of hydroxy group(s), particularly at the position 3′ of quercetin and myricetin, may play a crucial role in maintaining the B ring within the active site pocket of ENPP1. Thus, the remarkable inhibitory effects of quercetin and myricetin reported herein may explain their superior health benefits compared to other flavonoids^58–62^ involved in purinergic signaling pathways, such as morin and kaempferol. Future studies could consider investigating the effects of quercetin and myricetin on ENPP1 when using substrates associated with disease pathogenesis, such as cGAMP, which is linked to cancer.

### Supplementary Information

Supplementary Information.

## Data Availability

The datasets used and/or analyzed during the current study available from the corresponding author on reasonable request.

## References

[1] Giuliani AL, Sarti AC, Virgilio FD (2019). Extracellular nucleotides and nucleosides as signalling molecules. Immunol. Lett..

[2] Borza R, Salgado-Polo F, Moolenaar WH, Perrakis A (2022). Structure and function of the ecto-nucleotide pyrophosphatase/ phosphodiesterase (ENPP) family: Tidying up diversity. J. Biol. Chem..

[3] Lee S-Y, Müller CE (2017). Nucleotide pyrophosphatase/phosphodiesterase 1 (NPP1) and its inhibitors. Med. Chem. Commun..

[4] Lee S-Y (2017). Substrate-dependence of competitive nucleotide pyrophosphatase/phosphodiesterase1 (NPP1) inhibitors. Front. Pharmacol..

[5] Terkeltaub R (2006). Physiologic and pathologic functions of the NPP nucleotide pyrophosphatase/phosphodiesterase family focusing on NPP1 in calcification. Purinergic Signal..

[6] Rosenthal AK, Ryan LM (2016). Calcium pyrophosphate deposition disease. N. Engl. J. Med..

[7] Yongwattana N (2020). Fluorescence differentiation of ATP-related multiple enzymatic activities in synovial fluid as a marker of calcium pyrophosphate deposition disease using Kyoto Green. Molecules.

[8] Chu X (2023). Human antibodies targeting ENPP1 as candidate therapeutics for cancers. Front. Immunol..

[9] Wang X, Lu X, Yan D, Zhou Y, Tan X (2022). Development of novel ecto-nucleotide pyrophosphatase/phosphodiesterase 1 (ENPP1) inhibitors for tumor immunotherapy. Int. J. Mol. Sci..

[10] Jung JE (2022). Discovery of 3,4-dihydropyrimido[4,5-d]pyrimidin-2(1H)-one and 3,4-dihydropyrido[2,3-d]pyrimidin-2(1H)-one derivatives as novel ENPP1 inhibitors. Bioorg. Med. Chem. Lett..

[11] Gangar M (2022). Design, synthesis and biological evaluation studies of novel small molecule ENPP1 inhibitors for cancer immunotherapy. Bioorgan. Chem..

[12] Lu P (2023). Case report: A rare homozygous variation in the ENPP1 gene, presenting with generalized arterial calcification of infancy in a Chinese infant. Front. Cardiovasc. Med..

[13] Carozza JA (2020). Structure-aided development of small-molecule inhibitors of ENPP1, the extracellular phosphodiesterase of the immunotransmitter cGAMP. Cell Chem. Biol..

[14] Kawaguchi M (2019). Development of an ENPP1 fluorescence probe for inhibitor screening, cellular imaging, and prognostic assessment of malignant breast cancer. J. Med. Chem..

[15] Chang L (2014). Imidazopyridine- and purine-thioacetamide derivatives: Potent inhibitors of nucleotide pyrophosphatase/phosphodiesterase 1 (NPP1). J. Med. Chem..

[16] Song S, Shao Z (2022). From myricetin to the discovery of novel natural human ENPP1 inhibitors: A virtual screening, molecular docking, molecular dynamics simulation, and MM/GBSA study. Molecules.

[17] Taheri Y (2020). Myricetin bioactive effects: Moving from preclinical evidence to potential clinical applications. BMC Complement Med. Ther..

[18] Nutmakul T (2022). A review on benefits of quercetin in hyperuricemia and gouty arthritis. Saudi Pharm. J..

[19] Hollman PCH, Arts ICW (2000). Flavonols, flavones and flavanols—Nature, occurrence and dietary burden. J. Sci. Food Agric..

[20] Magar RT, Sohng JK (2020). A review on structure, modifications and structure activity relation of quercetin and its derivatives. J. Microbiol. Biotechnol..

[21] Zhang C, Wang R, Zhang G, Gong D (2018). Mechanistic insights into the inhibition of quercetin on xanthine oxidase. Int. J. Biol. Macromol..

[22] Namasivayam V, Lee S-Y, Müller CE (2017). The promiscuous ectonucleotidase NPP1: Molecular insights into substrate binding and hydrolysis. Biochim. Biophys. Acta.

[23] Ojida A, Takashima I, Kohira T, Nonaka H, Hamachi I (2008). Turn-on fluorescence sensing of nucleoside polyphosphates using a xanthene-based Zn(II) complex chemosensor. J. Am. Chem. Soc..

[24] Wongkongkatep J, Ojida A, Hamachi I (2017). Fluorescence sensing of inorganic phosphate and pyrophosphate using small molecular sensors and their applications. Top. Curr. Chem..

[25] Yongwattana N (2020). Fluorescence determination of soluble pyrophosphate levels in synovial fluid as a marker of pseudogout using middle point of quantification concept and molecular sensor. Sci. Asia.

[26] Srinarawat W (2022). Fluorescence identification of arthropathic calcium pyrophosphate single crystals using alizarin red S and xanthene dipicolylamine Zn^II^ complex. Analyst..

[27] Kato K (2012). Crystal structure of Enpp1, an extracellular glycoprotein involved in bone mineralization and insulin signaling. Proc. Natl. Acad. Sci. U. S. A..

[28] Shayhidin E (2015). Quinazoline-4-piperidine sulfamides are specific inhibitors of human NPP1 and prevent pathological mineralization of valve interstitial cells. Br. J. Pharmacol..

[29] Dennis ML (2020). Crystal structures of human ENPP1 in apo and bound forms. Acta Crystallogr. Sect. D Str. Bio..

[30] Nagao A, Seki M, Kobayashi H (1999). Inhibition of xanthine oxidase by flavonoids. Biosci. Biotechnol. Biochem..

[31] Melzig MF (1996). Inhibition of adenosine deaminase activity of aortic endothelial cells by selected flavonoids. Planta Med..

[32] Qin P, Li X, Yang H, Wang ZY, Lu D (2019). Therapeutic potential and biological applications of cordycepin and metabolic mechanisms in cordycepin-producing fungi. Molecules.

[33] Lee JB (2019). A novel nucleoside rescue metabolic pathway may be responsible for therapeutic effect of orally administered cordycepin. Sci. Rep..

[34] Bhatia NK, Tomar VR, Ishika Kishor S, Deep S (2022). Effect of pH and temperature on physicochemical properties, aggregation behaviour and degradation kinetics of quercetin and baicalein in nearly aqueous media. J. Mol. Liq..

[35] Kato K (2018). Structural insights into cGAMP degradation by Ecto-nucleotide pyrophosphatase phosphodiesterase 1. Nat. Commun..

[36] Guedes IA (2021). New machine learning and physics-based scoring functions for drug discovery. Sci. Rep..

[37] Trott O, Olson AJ (2010). AutoDock Vina: Improving the speed and accuracy of docking with a new scoring function, efficient optimization, and multithreading. J. Comput. Chem..

[38] Samuel G, Ulf R (2015). The MM/PBSA and MM/GBSA methods to estimate ligand-binding affinities. Expert Opin. Drug Discov..

[39] Wang E (2019). End-point binding free energy calculation with MM/PBSA and MM/GBSA: Strategies and applications in drug design. Chem. Rev..

[40] Kokh DB (2018). Estimation of drug-target residence times by τ-random acceleration molecular dynamics simulations. J. Chem. Theory Comput..

[41] Frisch, M. J. *et al*. Gaussian 09, Revision D.01. (Gaussian, Inc.: Wallingford, CT, USA, 2009).

[42] Waterhouse A (2018). SWISS-MODEL: Homology modelling of protein structures and complexes. Nucleic Acids Res..

[43] Bienert S (2017). The SWISS-MODEL Repository—New features and functionality. Nucleic Acids Res..

[44] Pettersen EF (2004). UCSF Chimera—A visualization system for exploratory research and analysis. J. Comput. Chem..

[45] Pettersen EF (2021). UCSF ChimeraX: Structure visualization for researchers, educators, and developers. Protein Sci..

[46] Salomon-Ferrer R, Case DA, Walker RC (2013). An overview of the Amber biomolecular simulation package. WIREs Comput. Mol. Sci..

[47] Wang J, Wolf RM, Caldwell JW, Kollman PA, Case DA (2004). Development and testing of a general amber force field. J. Comput. Chem..

[48] Tian C (2020). ff19SB: Amino-acid-specific protein backbone parameters trained against quantum mechanics energy surfaces in solution. J. Chem. Theory Comput..

[49] Izadi S, Anandakrishnan R, Onufriev AV (2014). Building water models: A different approach. J. Phys. Chem. Lett..

[50] Darden T, York D, Pedersen L (1993). Particle mesh Ewald: An N⋅log(N) method for Ewald sums in large systems. J. Chem. Phys..

[51] Ryckaert JP, Ciccotti G, Berendsen HJ (1977). Numerical integration of the Cartesian equations of motion of a system with constraints: Molecular dynamics of n-alkanes. J. Comput. Phys..

[52] Berendsen HJ, Postma J, van Gunsteren WF, DiNola A, Haak J (1984). Molecular dynamics with coupling to an external bath. J. Chem. Phys..

[53] Uberuaga B, Anghel M, Voter A (2004). Synchronization of trajectories in canonical molecular-dynamics simulations: Observation, explanation, and exploitation. J. Chem. Phys..

[54] Nutho B (2022). Discovery of C-12 dithiocarbamate andrographolide analogues as inhibitors of SARS-CoV-2 main protease: In vitro and in silico studies. Comput. Struct. Biotechnol. J..

[55] Roe DR, Cheatham TE (2013). PTRAJ and CPPTRAJ: Software for processing and analysis of molecular dynamics trajectory data. J. Chem. Theory Comput..

[56] Miller BR, McGee TD, Swails JM, Homeyer N, Gohlke H, Roitberg AE (2012). MMPBSA.py: An efficient program for end-state free energy calculations. J. Chem. Theory Comput..

[57] Sun H (2018). Assessing the performance of MM/PBSA and MM/GBSA methods. 7. Entropy effects on the performance of end-point binding free energy calculation approaches. Phys. Chem. Chem. Phys..

[58] Ota H, Kodama A (2022). Dasatinib plus quercetin attenuates some frailty characteristics in SAMP10 mice. Sci. Rep..

[59] Zhao Z (2022). Reveals of quercetin’s therapeutic effects on oral lichen planus based on network pharmacology approach and experimental validation. Sci. Rep..

[60] Kannan S, Balakrishnan J, Govindasamy A, Arunagiri R (2022). New insights into the antibacterial mode of action of quercetin against uropathogen *Serratia marcescens* in-vivo and in-vitro. Sci. Rep..

[61] Chellian J, Mak KK, Chellappan DK, Krishnappa P, Pichika MR (2022). Quercetin and metformin synergistically reverse endothelial dysfunction in the isolated aorta of streptozotocin-nicotinamide-induced diabetic rats. Sci. Rep..

[62] Di Pierro F (2023). Quercetin as a possible complementary agent for early-stage COVID-19: Concluding results of a randomized clinical trial. Front. Pharmacol..

