# Supplementary material for: Naturally occurring quercetin and myricetin as potent inhibitors for human ectonucleotide pyrophosphatase/phosphodiesterase 1
Source: Sci Rep. 2024 Jan 2;14:125. doi: 10.1038/s41598-023-50590-7 (PMC10761680; doi:10.1038/s41598-023-50590-7)
Supplement: Supplementary file 1 — Supplementary Information. [file 41598_2023_50590_MOESM1_ESM.pdf]

Supplementary Information

Naturally occurring quercetin and myricetin as potent inhibitors for human ectonucleotide pyrophosphatase/phosphodiesterase 1

Peeradon Duangiad[1], Bodee Nutho[2], Thawatchai Chaijarasphong[1], Noppawan Phumala Morales[2], Thunyarat Pongtharangkul[1], Itaru Hamachi[3], Akio Ojida[4] and Jirarut Wongkongkatep[1,*]

[1]Department of Biotechnology, Faculty of Science, Mahidol University, Rama 6 Road, Bangkok 10400, Thailand

[2]Department of Pharmacology, Faculty of Science, Mahidol University, Rama 6 Road, Bangkok 10400, Thailand

[3]Department of Synthetic Chemistry and Biological Chemistry, Graduate School of Engineering, Kyoto University, Katsura, Nishikyo-ku, Kyoto 615-8510 Japan

[4]Graduate School of Pharmaceutical Sciences, Kyushu University, 3-1-1, Maidashi, Higashi-ku, Fukuoka 812-8582, Japan

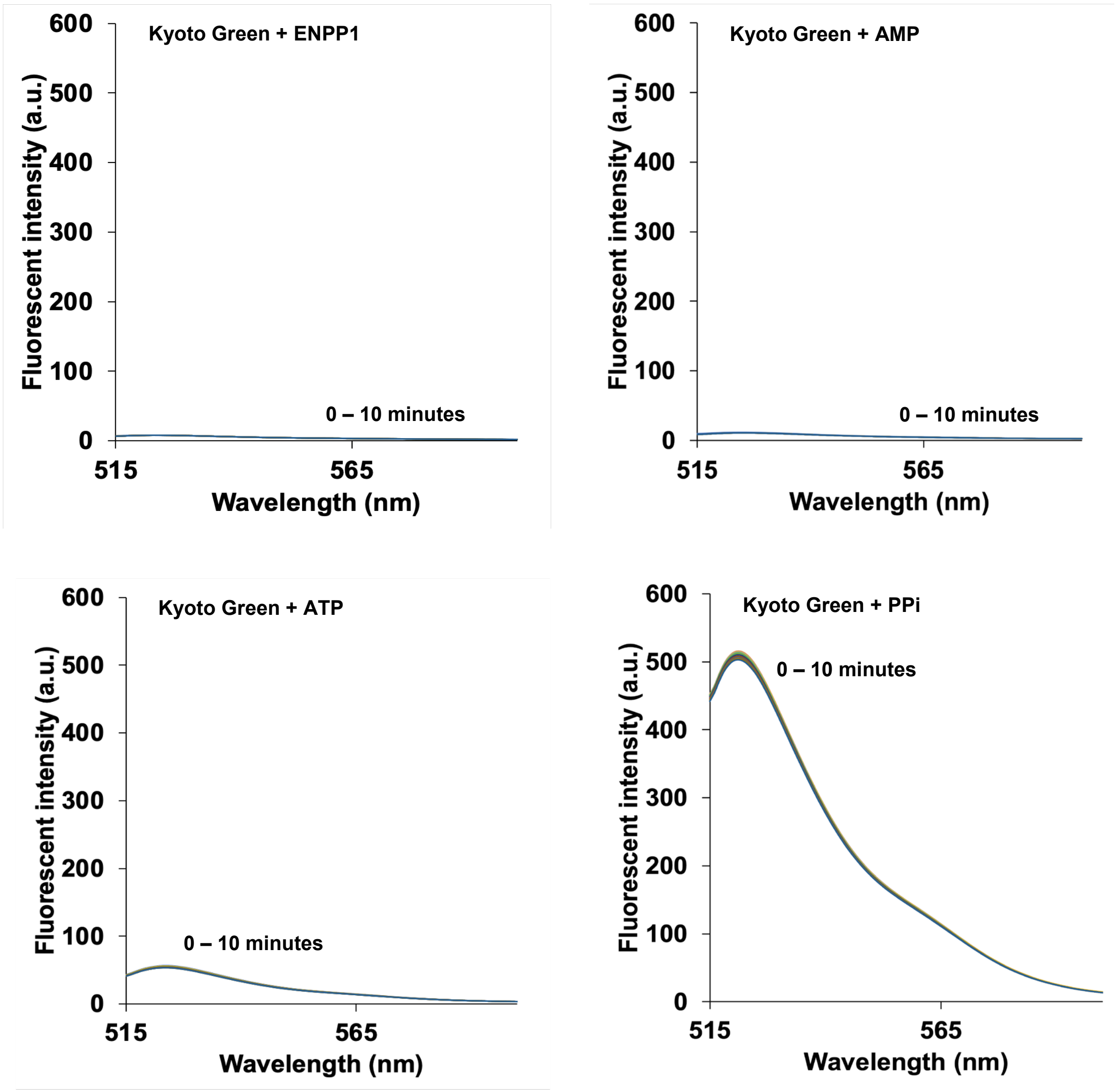

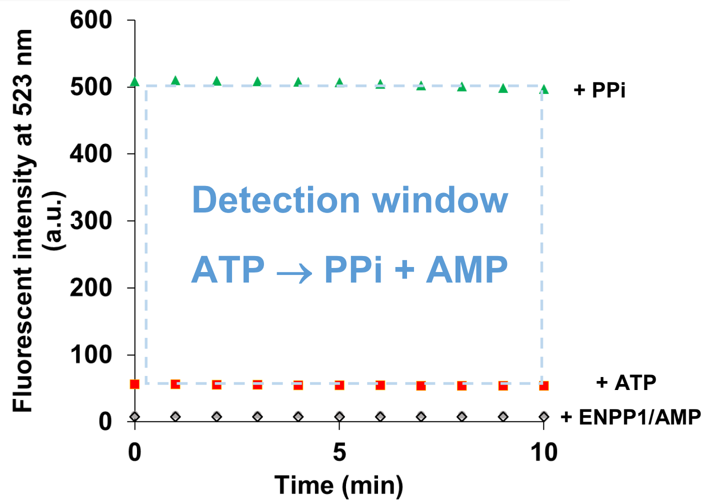

Figure S1. Stability of Kyoto Green during assay period. Fluorescence emission of Kyoto Green (1 μM) upon addition of ENPP1 (3.2 nM), AMP (1 μM), ATP (1 μM) and inorganic pyrophosphate (PPi, 1 μM) recorded at 1 min interval for 10 min. Measurement condition: 50 mM HEPES buffer containing 10 mM NaCl, 1 mM $MgCl_2$, 20 μM $Zn(NO_3)_2$ (pH 7.4, excitation wavelength = 488 nm)

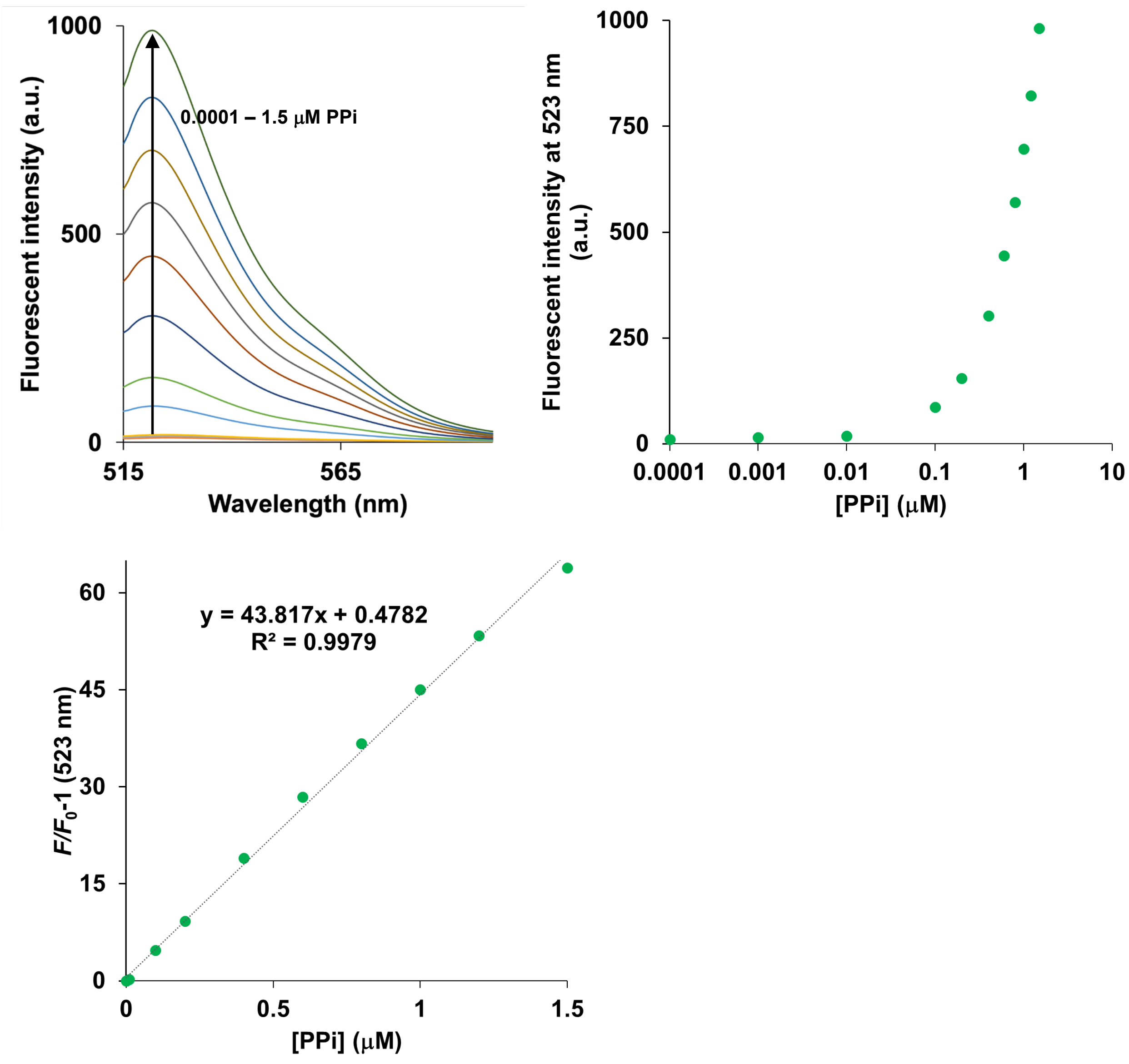

Figure S2. (top left) Fluorescence emission of Kyoto Green (1 μM) upon addition of inorganic pyrophosphate (PPi) and (top right) the relationship between the fluorescent intensity and concentration of PPi in logarithmic scale. (bottom) The relationship between fluorescence change (F/F_0-1) and PPi concentration. Measurement condition: 50 mM HEPES buffer containing 10 mM NaCl, 1 mM $MgCl_2$, 20 μM $Zn(NO_3)_2$ (pH 7.4, excitation wavelength = 488 nm)

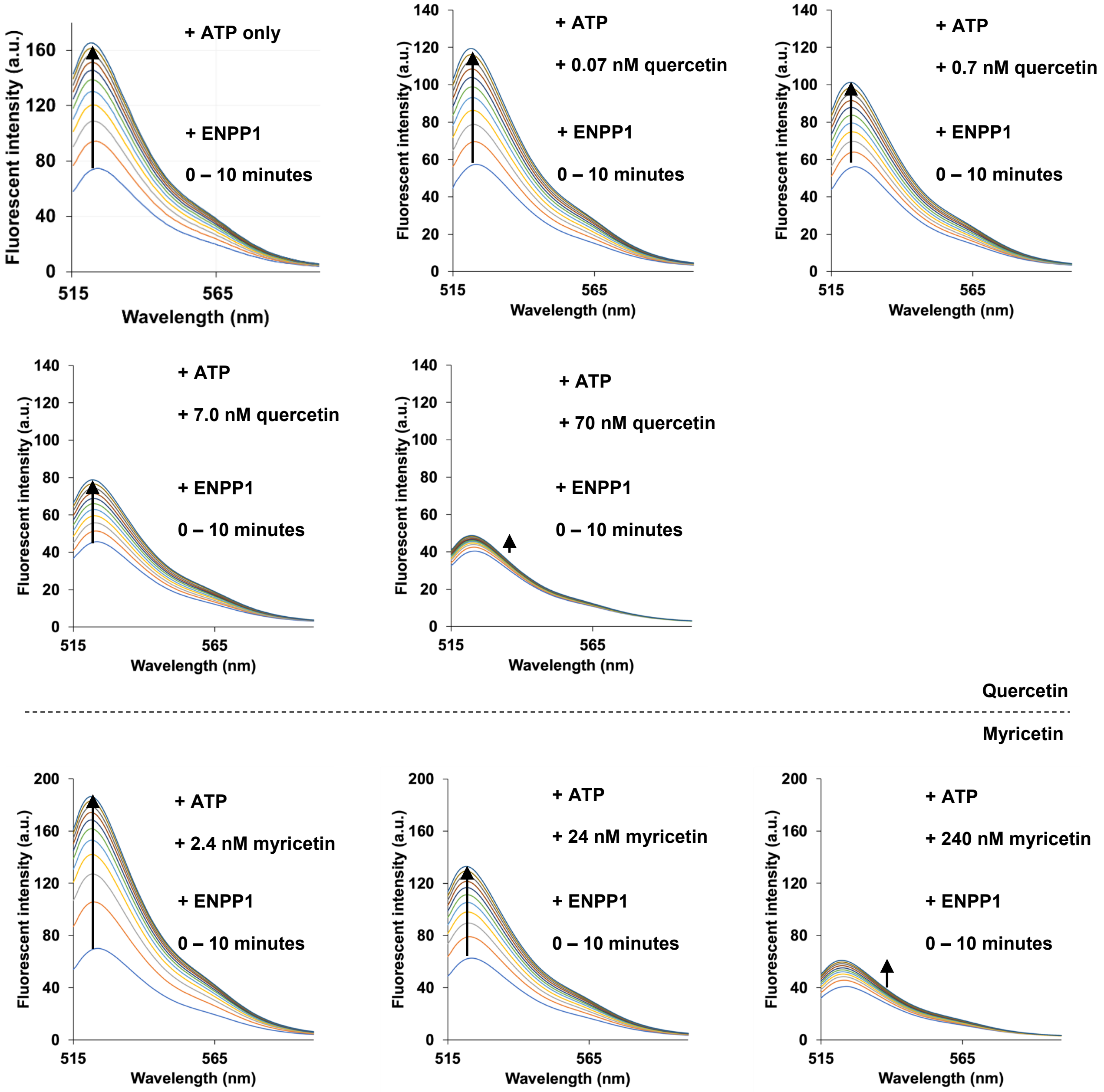

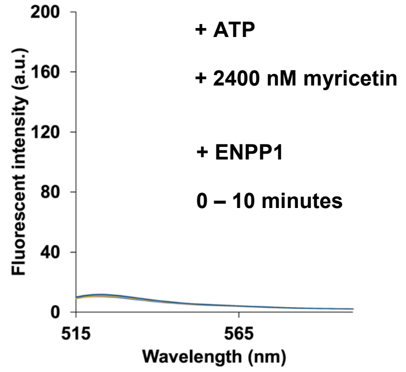

Figure S3. Fluorescence emission of Kyoto Green (1 μM) when incubated with ENPP1 (3.2 nM), ATP (1 μM) and various concentration of quercetin and myricetin. Assay condition: 50 mM HEPES buffer containing 10 mM NaCl, 1 mM $MgCl_2$, 20 μM $Zn(NO_3)_2$ (pH 7.4, excitation wavelength = 488 nm). F_0 = Fluorescent intensity of Kyoto Green after addition of ATP and ENPP 1 at 0 min.

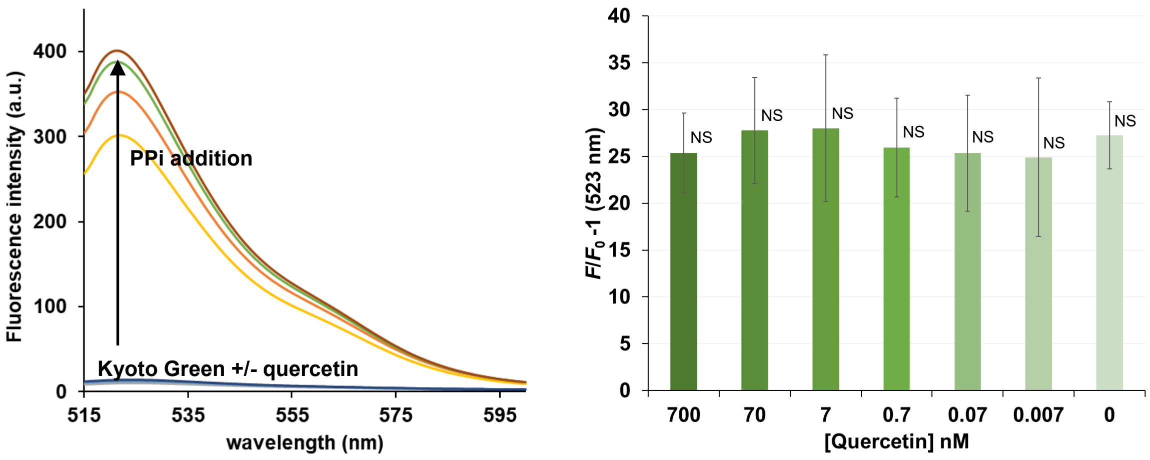

Figure S4. Effect of quercetin on the sensing capability of Kyoto Green for inorganic pyrophosphate. (Left) Fluorescence emission (JASCO FP6500, Japan) of Kyoto Green (1 μM) upon the addition of inorganic pyrophosphate (PPi, 1 μM) in the presence of quercetin at concentration of 0 (orange), 7 (brown), 70 (green) and 700 nM (yellow). (Right) Change in fluorescence emission of Kyoto Green (1 μM) in the presence of (PPi, 1 μM) and various concentrations of quercetin. NS: not significantly different (Tukey's test, $p > 0.05$). Assay condition: 50 mM HEPES buffer containing 10 mM NaCl, 1 mM $MgCl_2$, 20 μM $Zn(NO_3)_2$ (pH 7.4). Fluorescence emission at 523 nm (excitation wavelength = 488 nm) in the presence and absence of PPi was denoted as F and F_0, respectively.

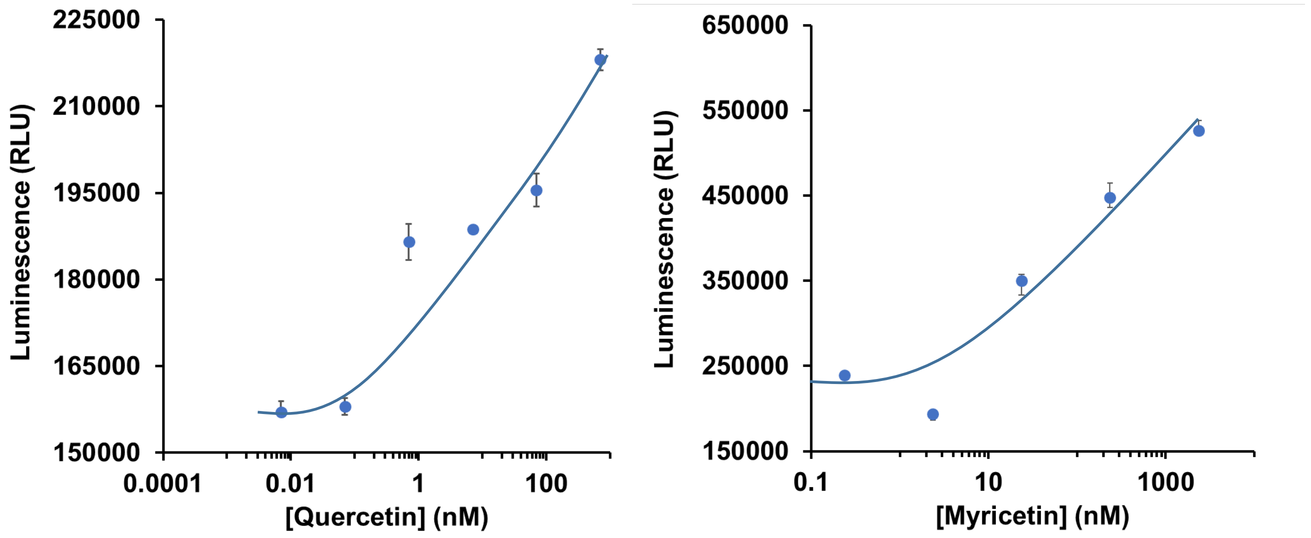

Figure S5. Luminescence ENPP1 assay. Hydrolytic reaction of ATP catalyzed by ENPP1 evaluated by ENLITEN® ATP assay system bioluminescence detection kit (Promega, USA) in the presence of various concentration of quercetin (left) and myricetin (right), n=3.

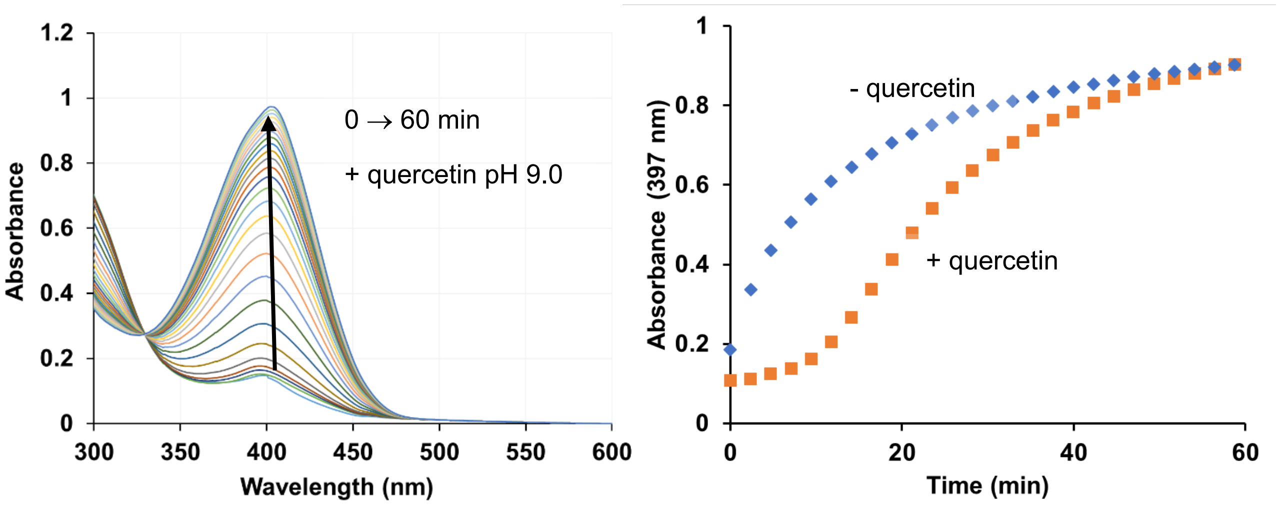

Figure S6. Real-time colorimetric ENPP1 assay. (Left) Time course of UV-visible spectrum (JASCO V730, Japan). (Right) Change of absorbance at 397 nm during hydrolysis of *p*-nitrophenyl 5′-thymidine monophosphate (*p*-Nph-5′-TMP, 0.1 mM) catalyzed by ENPP1 (6.5 nM) in the absence (blue diamond) and presence of quercetin (700 nM, orange square). The reaction contained 50 mM borate buffer (pH 9.0) containing 10 mM NaCl, 1 mM $MgCl_2$, 0.02 mM $Zn(NO_3)_2$. Measurements were performed at 25±2 °C.

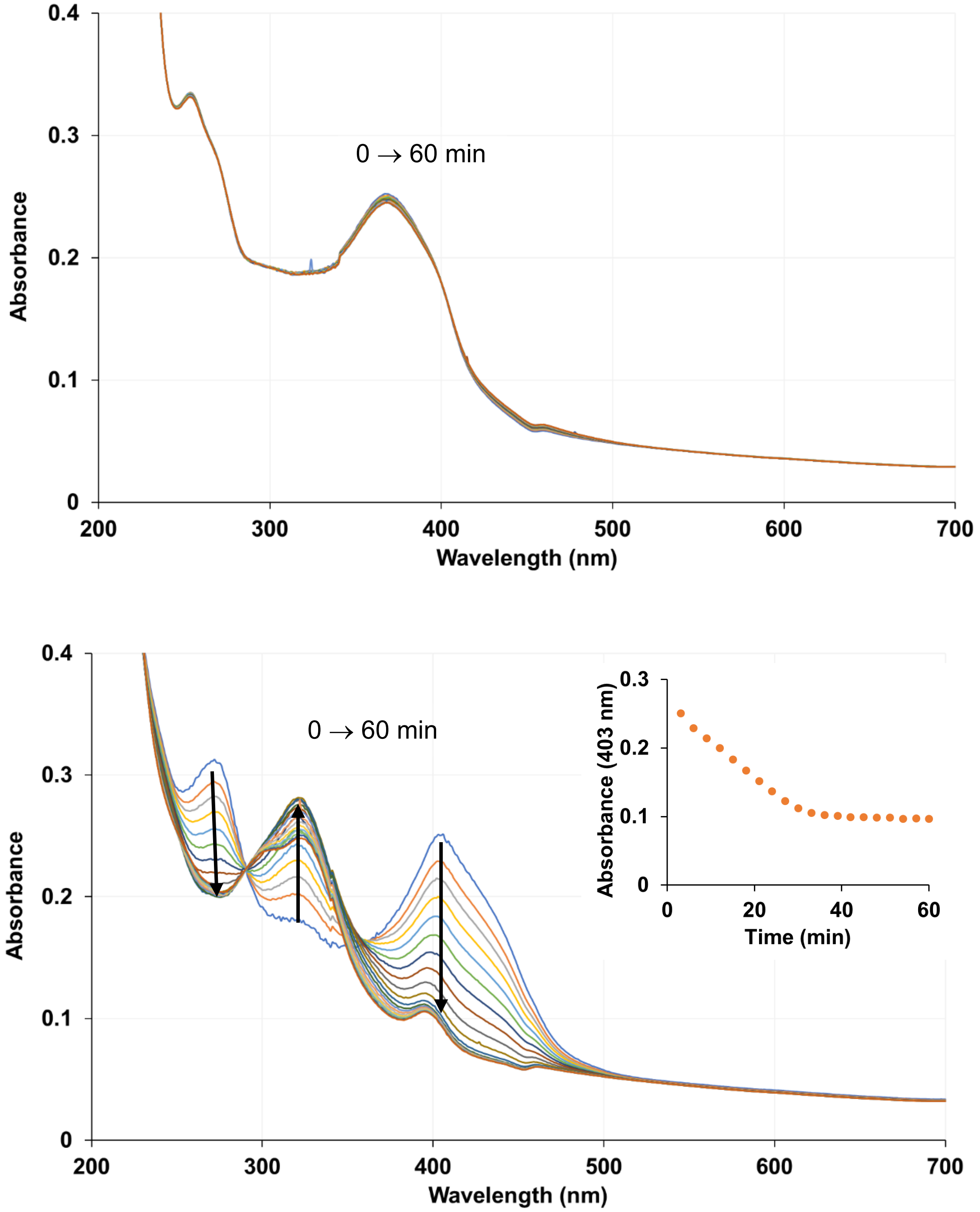

Figure S7. pH-dependent stability of quercetin in aqueous solution. Time course of UV-visible spectrum (JASCO V730, Japan) of quercetin (7 μM) in 50 mM HEPES buffer (pH 7.4) (above) or 50 mM borate buffer (pH 9.0) (below). Inset: Absorbance at λ_{max} (403 nm). Ratio of ethanol : buffer = 1 : 10 v/v. The buffer contained 10 mM NaCl, 1 mM $MgCl_2$, and 0.02 mM $Zn(NO_3)_2$. Measurements were performed at 25±2 °C.

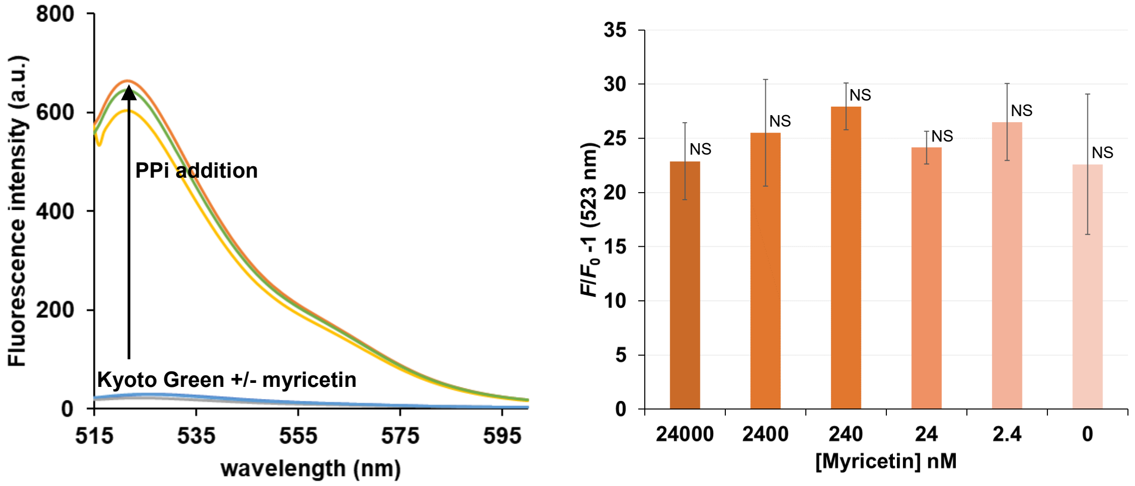

Figure S8. Effect of myricetin on the sensing capability of Kyoto Green for inorganic pyrophosphate. (Left) Fluorescence emission (JASCO FP6500, Japan) of Kyoto Green (1 μM) upon the addition of inorganic pyrophosphate (PPi, 1 μM) in the presence of myricetin at concentrations of 0 (orange), 2400 (green), and 24,000 nM (yellow). (Right) Change in fluorescence emission of Kyoto Green (1 μM) in the presence of PPi (1 μM) and various concentrations of quercetin. NS: not significantly difference (Tukey's test, $p > 0.05$). Assay condition: 50 mM HEPES buffer containing 10 mM NaCl, 1 mM $MgCl_2$, 20 μM $Zn(NO_3)_2$ (pH 7.4), temperature of 25±2 °C. Fluorescence emission at 523 nm (excitation wavelength = 488 nm) in the presence and absence of PPi was denoted as F and F_0, respectively.

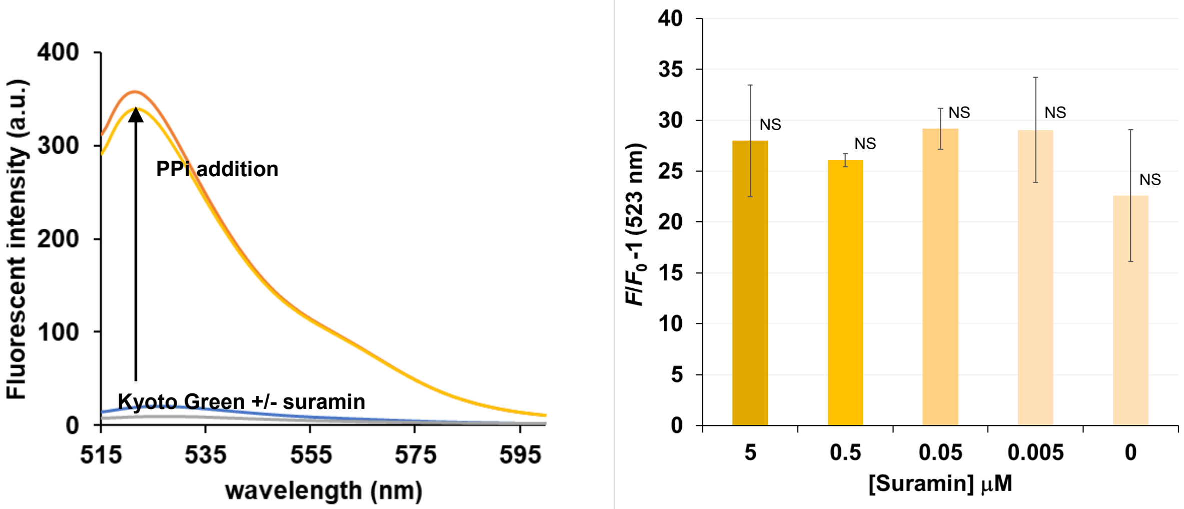

Figure S9. Effect of suramin on the sensing capability of Kyoto Green for inorganic pyrophosphate. (Left) Fluorescence emission (JASCO FP6500, Japan) of Kyoto Green (1 μM) upon the addition of inorganic pyrophosphate (PPi, 1 μM) in the presence of suramin at concentrations of 0 (orange) and 5 μM (yellow). (Right) Change in fluorescence emission of Kyoto Green (1 μM) in the presence of PPi (1 μM) and various concentrations of suramin. NS: not-significantly difference (Tukey's test, $p > 0.05$). Assay condition: 50 mM HEPES buffer containing 10 mM NaCl, 1 mM $MgCl_2$, 20 μM $Zn(NO_3)_2$ (pH 7.4), temperature of 25±2 °C. Fluorescence emission at 523 nm (excitation wavelength = 488 nm) in the presence and absence of PPi was denoted as F and F_0, respectively.

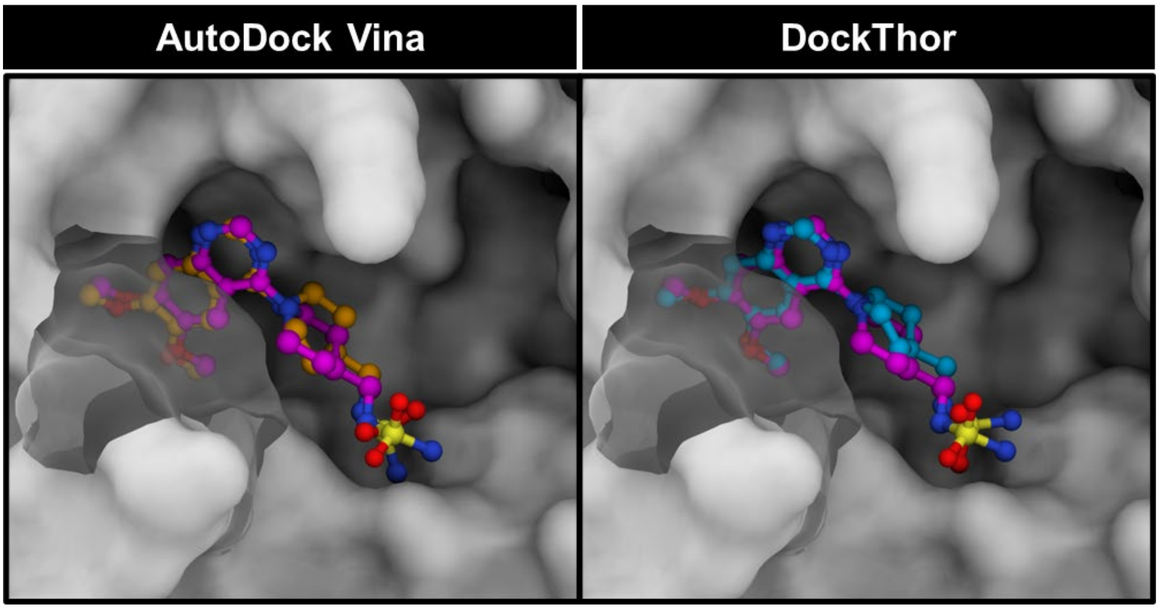

Figure S10. Superposition of the co-crystallized inhibitor **4** (magenta) with its redocked pose at the human ENPP1 active site, derived from AutoDock Vina (orange) and DockThor (light blue) docking programs. Red, yellow and blue atoms denote oxygen, sulfur and nitrogen atoms, respectively.

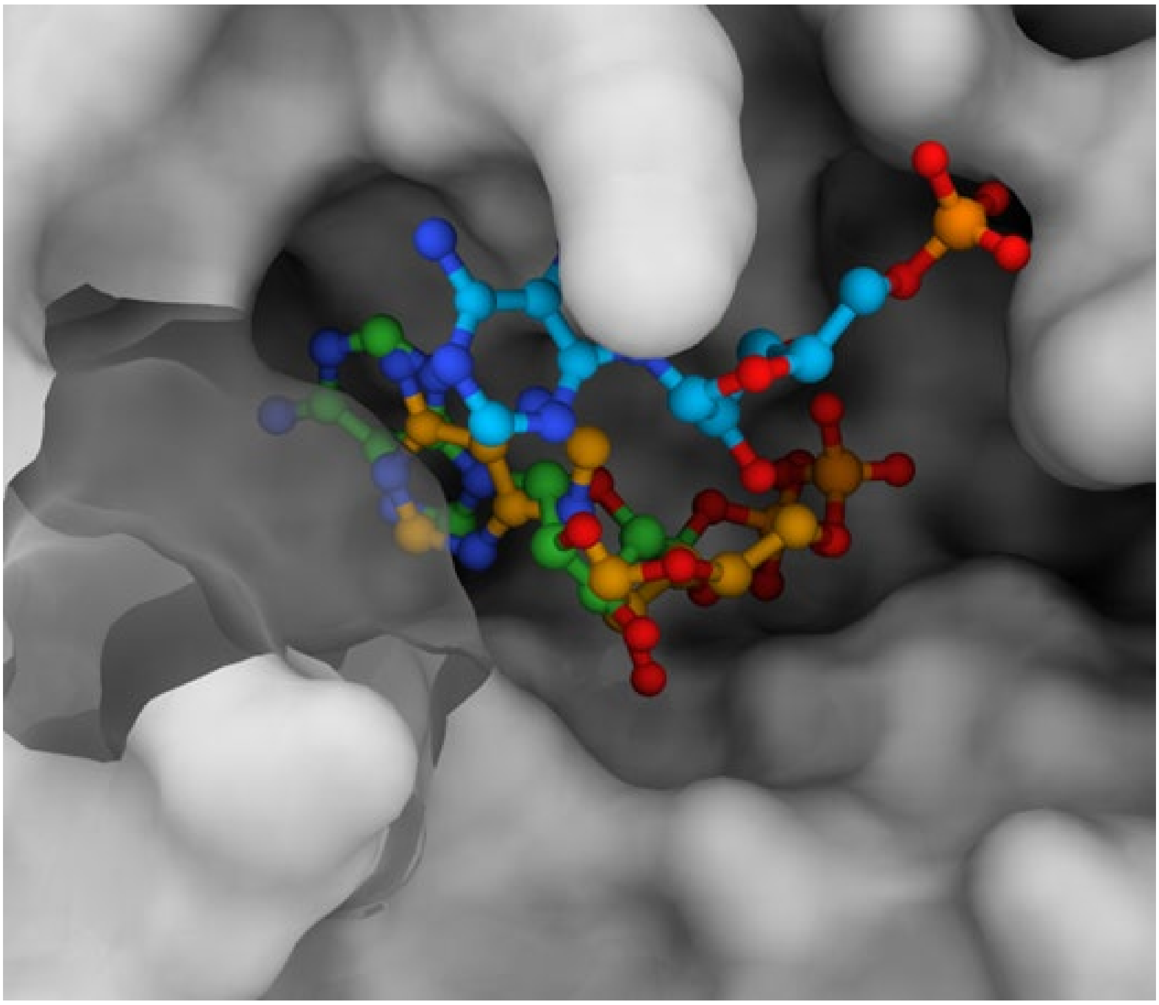

Figure S11. Superposition of the docked pose of AMP at the human ENPP1 active site derived from AutoDock Vina (orange) and DockThor (light blue) docking programs compared to the crystallographic pose (green) of the human ENPP1–AMP complex (PDB ID: 6WFJ available from Dennis, M. L. *et al*. Crystal structures of human ENPP1 in apo and bound forms. *Acta Crystallogr. Sect. D Str. Bio.* **76**, 889-898 (2020)).

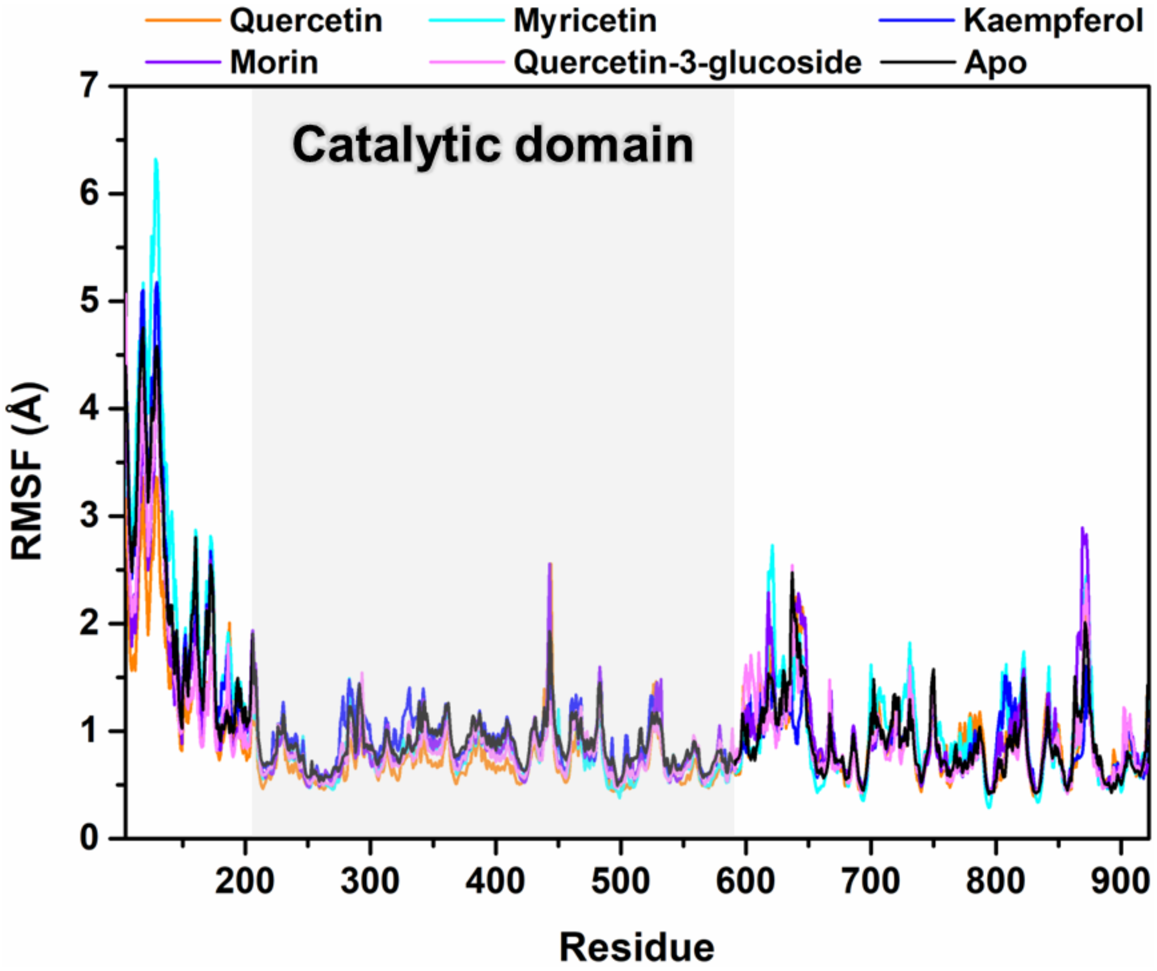

Figure S12. Plot of root means square fluctuations (RMSF) for C_α atoms of human ENPP1 in complex with different ligands: quercetin (orange), myricetin (cyan), kaempferol (blue), morin (purple), quercetin-3-glucoside (magenta) in comparison to apo-form (black).

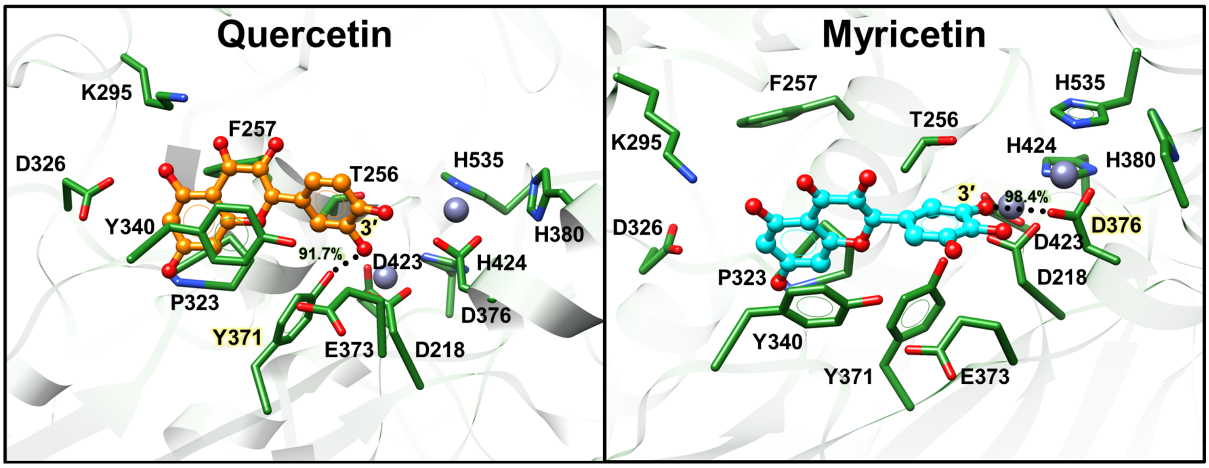

Figure S13. Results of MD simulations. Representative 3D structures of quercetin and myricetin bound in the active site of human ENPP1, showing a H-bond (black dotted lines) between the 3′-OH position of B ring and Y371 (for quercetin) or D376 (for myricetin).

Table S1 An expanded table showing the K_i values of quercetin, myricetin, and a selection of previously reported inhibitors for inhibition of human soluble ENPP1.

Compounds	Substrates	Methods (Reporters)	pH	K_i (nM)	References
Quercetin	ATP	Fluorescence (Kyoto Green)	7.4	4.0±0.8	This study
	ATP	Bioluminescence (Luciferin/luciferase)	7.4	4.9±0.5	This study
	p-Nph-5′-TMP	Colorimetry (*p*-nitrophenol)	7.4	159±73	This study
Myricetin	ATP	Fluorescence (Kyoto Green)	7.4	32±2.2	This study
	ATP	Bioluminescence (Luciferin/luciferase)	7.4	5.1±0.6	This study
	p-Nph-5′-TMP	Colorimetry (*p*-nitrophenol)	7.4	116±24	This study
	p-Nph-5′-TMP	Colorimetry (*p*-nitrophenol)	7.4	(432)	[16]
	p-Nph-5′-TMP	Colorimetry (*p*-nitrophenol)	9.5	(414)	
Suramin	ATP	Fluorescence (Kyoto Green)	7.4	753±55	This study
	ATP	Capillary Electrophoresis	9.0	780	[4]
	p-Nph-5′-TMP	Colorimetry (*p*-nitrophenol)	7.4	260±25	This study
	p-Nph-5′-TMP	Colorimetry (*p*-nitrophenol)	9.0	1070	[4]
**1**	ATP	Colorimetry (Molybdenum-phosphate complex)	9.0	2000	[14]
	p-Nph-5′-TMP	Colorimetry (*p*-nitrophenol)	9.0	480	[14]
**2**	ATP	Capillary Electrophoresis	9.0	5340	[15]
	p-Nph-5′-TMP	Colorimetry (*p*-nitrophenol)	9.0	29.6	[15]
**3**	ATP	Capillary Electrophoresis	9.0	18000	[15]
	p-Nph-5′-TMP	Colorimetry (*p*-nitrophenol)	9.0	5	[15]
**4**	ATP	Capillary Electrophoresis	9.0	215	[4]
	p-Nph-5′-TMP	Colorimetry (*p*-nitrophenol)	9.0	64.2	[4]
**5**	cGAMP	Bioluminescence (Enzyme-coupling)	7.4	< 2	[13]
**6**	cGAMP	Bioluminescence (Enzyme-coupling)	8.5	(3.7)	[10]
**7**	cGAMP	Bioluminescence (Enzyme-coupling)	9.5	2.9	[11]
	p-Nph-5′-TMP	Colorimetry (*p*-nitrophenol)	9.0	41	

Values in parentheses indicate IC_{50}, provided as substitutes for K_i when the latter is not available.

Table S2. Docking scores between each compound and human ENPP1 as determined from AutoDock Vina and DockThor programs.

**Compounds**	**Binding energy (kcal/mol)**	
	**AutoDock Vina**	**DockThor**
Quercetin	–8.86	–8.73
Myricetin	–8.82	–8.75
Kaempferol	–8.55	–8.69
Morin	–8.84	–8.49
Quercetin-3-glucoside	–8.81	–9.28
Quinazoline (compound **4**)	–9.19	–9.40
AMP	–7.41	–6.97
